# Involvement of a citrus meiotic recombination TTC-repeat motif in the formation of gross deletions generated by ionizing radiation and MULE activation

**DOI:** 10.1186/s12864-015-1280-3

**Published:** 2015-02-13

**Authors:** Javier Terol, Victoria Ibañez, José Carbonell, Roberto Alonso, Leandro H Estornell, Concetta Licciardello, Ivo G Gut, Joaquín Dopazo, Manuel Talon

**Affiliations:** Centro de Genómica, Instituto Valenciano de Investigaciones Agrarias (IVIA), Moncada, 46113 Valencia Spain; Centro de Investigación Principe Felipe (CIPF), Avda, Autopista del Saler, 16-3, 46012 Valencia, Spain; CRA-ACM, Consiglio per la Ricerca e la Sperimentazione in Agricoltura, Corso Savoia 190, 95024 Acireale, Catania Italy; Centro Nacional de Análisis Genómico, Parc Científic de Barcelona, 08028 Barcelona, Spain

**Keywords:** Double-strand breaks, Crossover hot spot, Structural variations, Transposable-element

## Abstract

**Background:**

Transposable-element mediated chromosomal rearrangements require the involvement of two transposons and two double-strand breaks (DSB) located in close proximity. In radiobiology, DSB proximity is also a major factor contributing to rearrangements. However, the whole issue of DSB proximity remains virtually unexplored.

**Results:**

Based on DNA sequencing analysis we show that the genomes of 2 derived mutations, Arrufatina (sport) and Nero (irradiation), share a similar 2 Mb deletion of chromosome 3. A 7 kb Mutator-like element found in Clemenules was present in Arrufatina in inverted orientation flanking the 5′ end of the deletion. The Arrufatina Mule displayed “dissimilar” 9-bp target site duplications separated by 2 Mb. Fine-scale single nucleotide variant analyses of the deleted fragments identified a TTC-repeat sequence motif located in the center of the deletion responsible of a meiotic crossover detected in the citrus reference genome.

**Conclusions:**

Taken together, this information is compatible with the proposal that in both mutants, the TTC-repeat motif formed a triplex DNA structure generating a loop that brought in close proximity the originally distinct reactive ends. In Arrufatina, the loop brought the Mule ends nearby the 2 distinct insertion target sites and the inverted insertion of the transposable element between these target sites provoked the release of the in-between fragment. This proposal requires the involvement of a unique transposon and sheds light on the unresolved question of how two distinct sites become located in close proximity. These observations confer a crucial role to the TTC-repeats in fundamental plant processes as meiotic recombination and chromosomal rearrangements.

**Electronic supplementary material:**

The online version of this article (doi:10.1186/s12864-015-1280-3) contains supplementary material, which is available to authorized users.

## Background

One of the major lines of evidence supporting that structural variations in genomes have a strong impact on phenotypic diversity comes from the study of human genomes (www.1000genomes.org/) and their prevalence on diseases [1]. It is well known that structural genome variations may occur through numerous processes, i.e. segmental duplications, illegitimate recombination or transposable elements (TEs) activity [1,2]. TE insertions specially provide an extraordinary source of natural genetic variation and diversity [3]. Transposable elements [4,5], as ionizing radiation [6-8] for instance, have frequently been associated with major chromosomal rearrangements such as deletions, duplications, inversions, translocations and recombination of host genomes [4,5].

In previous work, we generated through irradiation of *Citrus clementine*, cv. “Clemenules”, (CLE) a collection of induced mutants in order to increase phenotypic diversity. The screening for fruit precocity of this collection identified a mutant, Nero (NER), strongly resembling the spontaneous Arrufatina somatic mutation. Since ionizing radiation is generally expected to produce mostly deletions [6] it was hypothesized that a similar deletion rearrangement might be the cause of the precocious behavior of the ARR natural mutation. In the work presented in here we took advantage of the availability of the citrus clementine genome (GenBank: AMZM00000000.1) to show that both mutants certainly share a similar 2 Mb deletion of chromosome 3 and that the ARR deletion is associated with the activation of a Mutator-like element (MULE). Mutator and MULEs are widespread in plants, fungi and animals. MULEs contain transposase domains, terminal inverted repeats (TIRs) and generally have a 9–11 bp target site duplication (TSD) flanking the transposon formed during “cut and paste” transposition [9] into a new genomic location [10-13]. In general, there is solid evidence showing that Mutator frequently induces deletions [14] and although major advances have been made in the biology of TEs, there are still many open questions to be elucidated on this association. According to Gray [15], there are 2 possible mechanisms by which TE-associated chromosomal rearrangements may occur: homologous recombination and alternative transposition process. During homologous recombination, sequences are exchanged between homologous DNA fragments. For instance, intra-strand homologous recombination between two different TEs may result in deletion of the in-between region. In alternative transposition a hybrid element is formed after the synapsis of complementary TE ends from separate TEs. Depending upon the orientation of the termini and on the chromosomal location of the elements, alternative transpositions can lead to many kinds of chromosomal rearrangements including inversions, duplications, and deletions [4]. It is well known that pairs of closely-linked transposable elements can induce various chromosomal rearrangements in several systems, through both, homologous recombination and alternative transposition [14,16-18]. Nevertheless, there are many examples where bimolecular synapsis cannot explain all TE-mediated rearrangements not resolved by homologous recombination [15]. In fact, the observed characteristics of the ARR deletion do not match the accepted premises of homologous recombination, alternative transposition either those of “cut and paste” transposition.

On the other hand, ionizing radiation of cells has provided a large body of evidence that chromosomal rearrangements are clearly influenced by “proximity” effects [19]. Illegitimate repair, for example, decreases as the distance between DSBs at the time of formation increases. Furthermore, the production of interstitial deletions is larger than randomness would indicate. Therefore, the occurrence of a gross deletion implies the presence of double-stranded breaks (DSBs) in two distinct genomic locations physically located in close proximity and joined by incorrect repair. However, it remains still very unclear how two genomically distinct sites become located in close proximity. For instance, Van Zelm et al. [18] studied gross deletions in human genes and found that the breakpoints analyzed involved at least two DSBs in distant genomic locations that were first placed in physical proximity and then incorrectly repaired. The authors performed a careful evaluation of the current hypothesis to explain this circumstance and conclude that unknown additional factors were required to mediate co-localization of two distant genomic regions and double-stranded DNA break induction. While these unknown factors have resulted rather elusive to date, in the current work we provide evidence compatible with the suggestion that a TTC-repeat motif very similar to the recently reported CTT-repeat DNA motif from an *Arabidopsis* meiotic crossover hot spot [20,21], may enable the proximity of the two distant sequences facilitating transposase reactions.

## Results and discussion

It is widely accepted that transposable-element mediated chromosomal rearrangements require coordinated transposition or involvement of at least two TEs. Thus, several mechanisms generating chromosomal rearrangements have been devised mostly based on variations of the basic homologous recombination and alternative transposition processes [4,15]. While these mechanisms have received wide experimental support, there are many examples where TE-mediated rearrangements are mostly incompatible with homologous recombination and with the classical or alternative “cut and paste” transposition [15]. In addition, the occurrence of gross or large rearrangements also implies the presence of DSBs in two distinct and separate genomic locations but physically located in close proximity to allow TE insertion. This question, how two distinct chromosomal sites become close together, is a current unresolved enigma attributed to unknown factors [18]. In the current work we provide evidence in citrus suggesting that a TTC-repeat motif of a meiotic crossover hot spot might enable the proximity of two distant sequences facilitating transposition of a TE that as a consequence generated a gross deletion.

In this work, we took advantage of the availability of the citrus clementine genome (GenBank: AMZM00000000.1) to identify and characterize a structural deletion involved in citrus fruit precociousness through the analysis and comparison of the genomes of three clementines (*Citrus clementine*), Clemenules (CLE), Arrufatina (ARR) and Nero (NER). Both ARR and NER are mutants derived from somatic CLE mutations; ARR is a spontaneous bud sport whereas NER is a fast neutron induced mutant. The mutants are phenotypically very alike except for the sterility developed in the induced mutant and both show fruit precocity when compared with CLE (Additional file 1: Table S1).

### Genome sequencing

Illumina pair-end, short read technology was used to sequence the genomes of CLE, ARR and NER. For mapping, the high quality genome sequence of a haploid clementine variety generated by the International Citrus Genome Consortium (GenBank: AMZM00000000.1) was used as reference. The sequencing, mapping and variant calling statistics presented in Additional file 2: Table S2 indicates that the genome sequences obtained were of high quality. More than 400 million reads were mapped in CLE and approximately a half of these in both mutants. Coverage of the CLE (69x), ARR (44x) and NER (39x) genomes was, therefore, relatively high with more than 81% of the genome sequences covered by at least 15 reads. CLE Illumina reads were submitted to the NCBI Sequence Read Archive with the experiment accession number SRX371962. ARR and NER sequences were submitted to the European Nucleotide Archive with the study accession number PRJEB5808.

### Variant calling: SNVs and indels

Variant calling was performed with GATK [22] in order to identify single nucleotide variants (SNVs) and small indels (in general, no more than 15 bp). As the reference sequence used for the analysis was a haploid genotype of CLE, the number of SNVs identified in the diploid CLE genotype (1,4 million) was slightly smaller than those found in ARR and NER. Between 12–14 thousand SNVs in each genome were detected as homozygous SNVs as related to the reference genome (Additional file 2: Table S2). A deeper insight in these data showed that most of the reads actually had either multiallelic positions or low frequencies for the reference allele and that only 30% were pure homozygous SNVs, suggesting that these could be Sanger errors in the reference genome. Multiallelic positions and low allelic frequencies are probably linked to the chimeric nature of these genotypes as explained below. The variant calling also revealed about 150 thousand heterozygous indels and another 10 thousand homozygous indels.

### Coverage, copy number variation, pair-end reads and PCR analyses: chromosomal rearrangements

**Figure 1:**
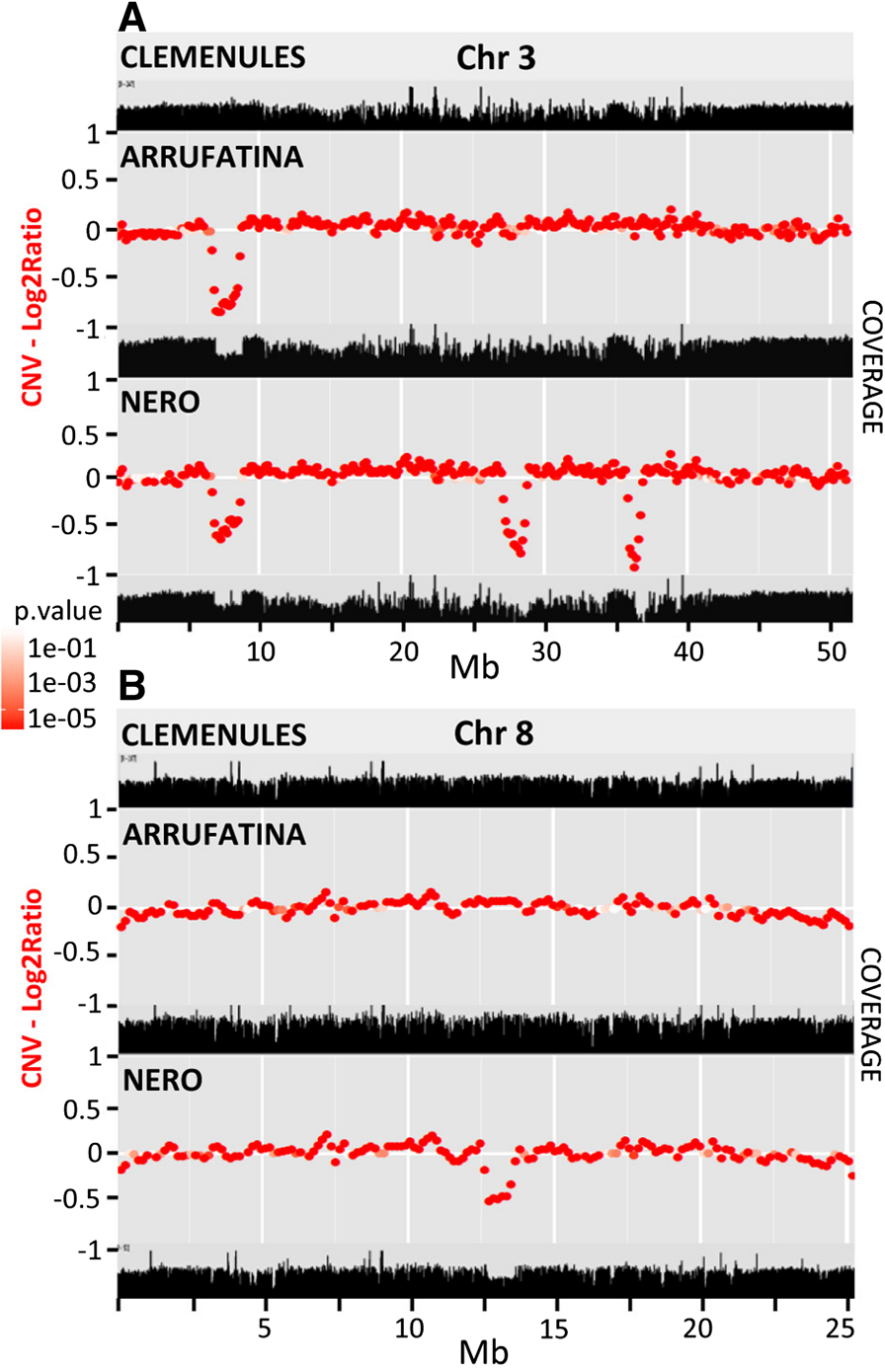
**Sequencing coverage and copy number variation (CNV).** The sequence coverage and the CNVs along chromosome 3 **(A)** and chromosome 8 **(B)** in three clementines, CLE, ARR and NER are shown. Read depths of each chromosome are depicted as black profiles in unitless scales. CNVs are shown as red points at a genome level log2 ratio between CLE, the original variety and either ARR or NER, the two mutations. The red color gradient sections represents log10 p calculated on each of ratios.

**Figure 2:**
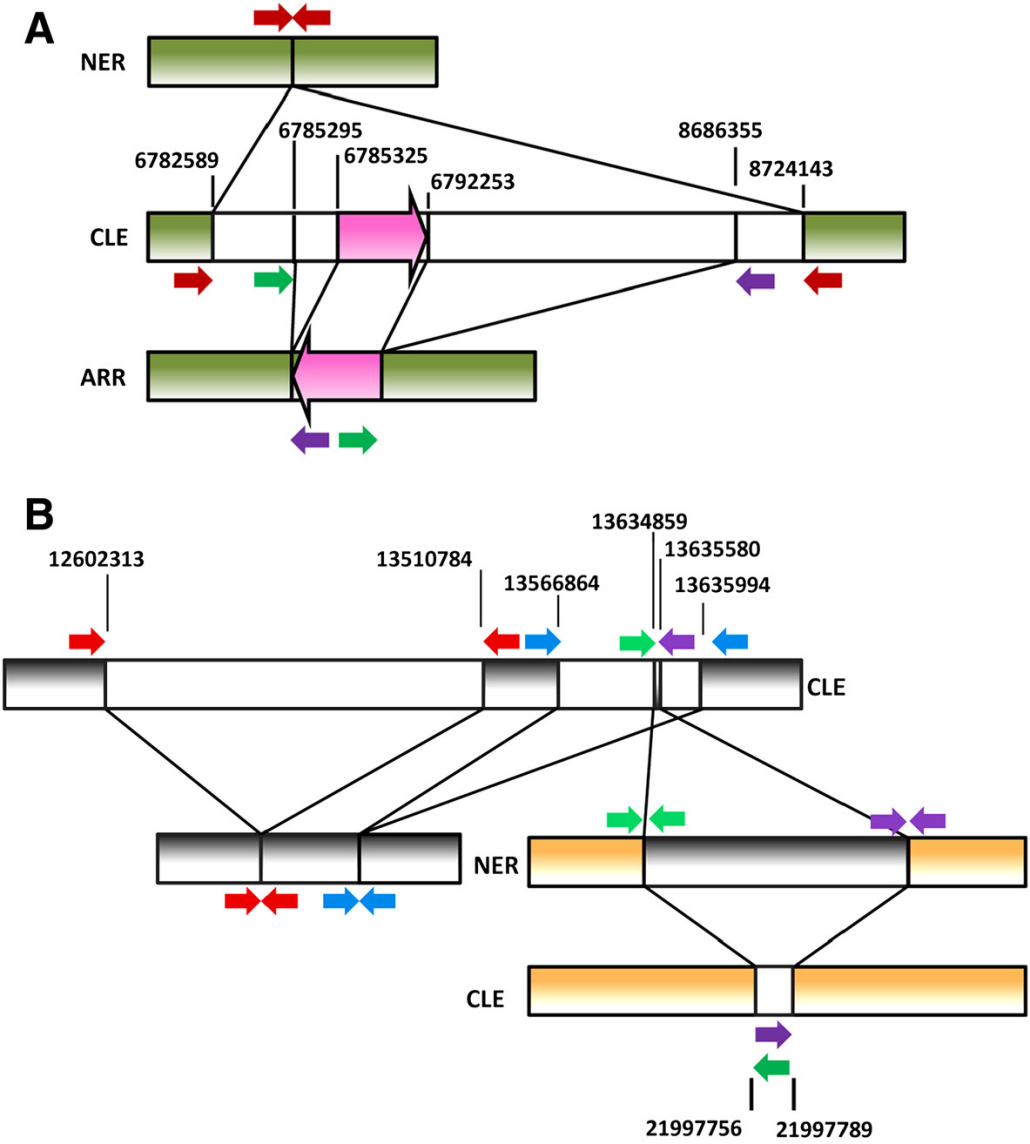
**Chromosomal rearrangements in ARR and NER. A)** Representation of the position and orientation of the pair-end reads (red, green and purple arrows) supporting the deletion of a similar fragment in chromosome 3 (green bars) of ARR and NER. Positions of breakpoints (bp) and the mapping of the pair reads are shown in the reference CLE genome. White bars flanking green bars represent fragments in CLE that are deleted in ARR and/or NER. The inversion found in ARR is shown as a pink big arrow. The different elements are not drawn to scale. **B)** Representation of the position and orientation of the pair-end reads (represented by colored arrows) supporting several rearrangements in chromosomes 8 (gray bars) and 6 (brown bars) of NER. Positions of breakpoints (bp) and the mapping of the pair reads are shown in the reference CLE genome. Red and blue pair-ends indicate the occurrence of two consecutive but separate deletions. Green and purple pair-ends support the occurrence of both a translocation from a small fragment of one of the initially deleted stretches of chromosome 8 to chromosome 6, and a small deletion in chromosome 6. White bars flanking either gray or brown bars represent fragments in CLE that are deleted in NER. The gray bar flanking brown bars represents the fragment in CLE that was translocated from chromosome 8 to chromosome 6. The different elements are not drawn to scale.

Chromosomal rearrangements were investigated through 4 independent analyses based on read depth, copy number variation, pair-end reads and PCR. Coverage profile for a certain chromosome was remarkably similar in the three genomes except in a few stretches of ARR and NER that showed reduced depth, suggesting the occurrence of putative gross deletions. Reduced coverage was observed in a very similar fragment of chromosome 3 in both mutants, while NER showed three additional putative interstitial deletions (Figure 1A) two of them also in chromosome 3 and the third one in chromosome 8 (Figure 1B). Copy number variation analysis with CNV-seq [23] confirmed the occurrence of the four deletions insinuated with the coverage reduction. Thus, the deletions predicted by coverage analysis and CNV-seq were completely coincident. Further pair-end read analyses provided additional insights on rearrangements of chromosomes 3 and 8 in both mutated genotypes (Additional file 3: Table S3). While the orientation of pair reads indicated the nature of the rearrangement (deletion vs. inversion or translocation) the read percentage suggested the hemizygous condition of the event. The read pairing analyses ascertained the presence of the intriguing deletion of chromosome 3 common to the two mutated genotypes, and the NER deletion of chromosome 8. However, there was no pair read evidence supporting the two additional NER deletions of chromosome 3 spanned from positions 27.2 to 28.7 and 36.1 to 37.0 MB (Figure 1A), probably because these were located in low complexity areas and the corresponding reads were filtered during mapping. In ARR, the paired-end analyses identified a 6926 bp inversion of chromosome 3 (Figure 2A) apparently supported by a percentage of pair-end reads lower than the expected 50%, as shown in Additional file 3: Table S3. The structure indicated by this pairing, however, was clearly confirmed by PCR. There were other 3 sets of pair-end reads involving 3 different positions at chromosome 4. These pairings probably were mapping artifacts since no PCR evidence of their occurrence was obtained. This observation, however, indicated that the inverted sequence was repeated at least 4-fold in the clementine genome and prompted the suggestion that it might be a repetitive sequence. It is also worth to note that the presence of this inversion implies the occurrence of a deletion spanning from positions 6785295 to 8686355, a stretch very similar although not identical to the deletion identified in NER that spanned from positions 6782589 to 8724143. According to the data, the NER deletion of chromosome 8 was actually a double interstitial deletion composed of a gross and a small deletion separated by about 50 thousand bp (Figure 2B). In addition, the pair-end analyses provided evidence for a 721 bp translocation from the smaller deletion of chromosome 8 to chromosome 6, an event that also resulted in a 33 bp deletion of chromosome 6 (Additional file 3: Table S3).

All boundaries of the above mentioned rearrangements were confirmed by PCR analyses except the 3 deletions of chromosome 3 from NER. The first deletion, however, was ascertained by gene dosage because the 5′ boundary of the deletion was resistant to amplification due to the occurrence of a 32 bp palindrome at the beginning of the deletion. On the other hand, precise boundaries for the other 2 deletions could not be well defined.

### MULE identification, characterization and phylogenetic analyses

**Figure 3:**
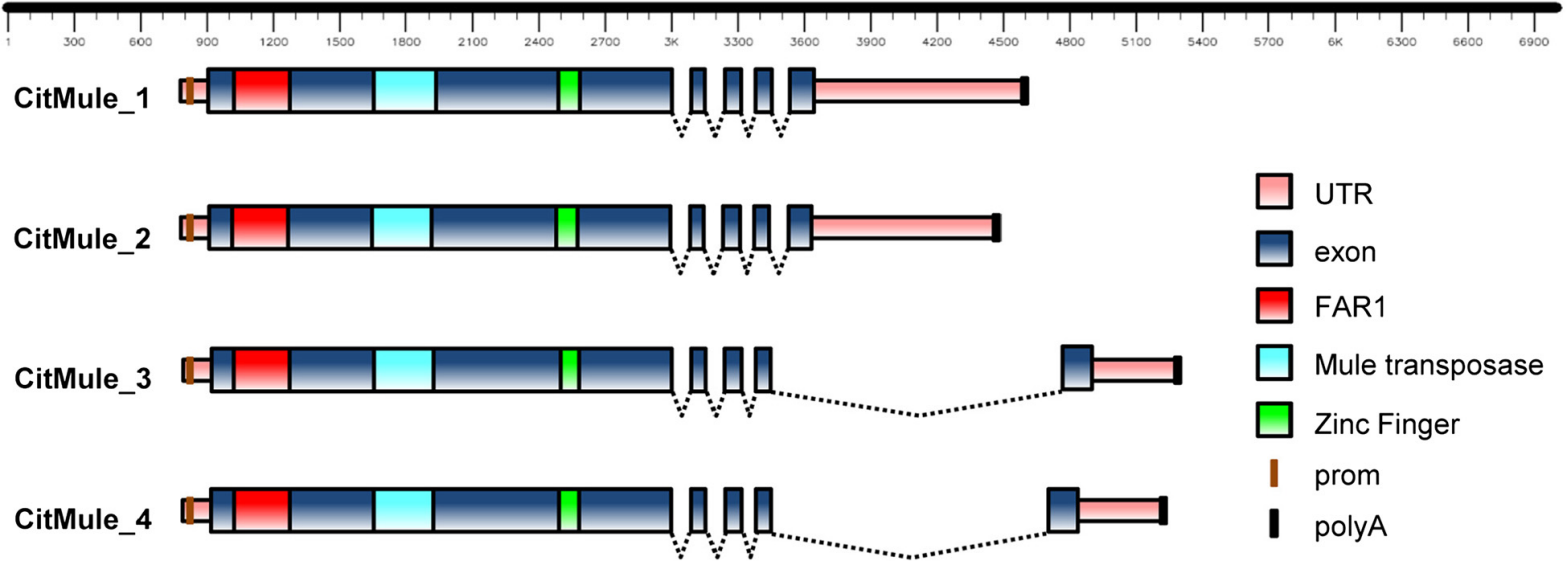
**Structure of 4 CitMul elements.** Transposable CitMul elements contain a 5 exon transcript, with a largest exon 1 including predicted protein sequences coding for a FAR1 DNA binding domain, a SWIM-type zinc finger motif and a MULE transposase domain. Introns are depicted as lines connecting exons.

Further characterization of the 6926 bp inverted stretch flanking the deletion of ARR chromosome 3 indicated that this sequence was representative of a family of Mutator-like elements (MULEs) named in here CitMule (Figure 3). A BLASTN search performed against the CLE reference genome, using 10 kb tracks at both sides of the 5′ breakpoint of the ARR deletion as queries, identified 3 regions on chromosome 4 with a sequence identity to the inverted fragment higher than 99%. A BLASTX search against the NCBI protein database showed significant similarity of this sequence with several members of the Mutator superfamily of transposable elements. In comparison with CitMul_1 that was taken as the reference sequence, CitMul_2 contained 2 small deletions at positions 2572–2656 (84 bp) and at positions 6637 to 6665 (8 bp). Similarly, CitMul_4 (6864 bp) contained 3 deletions at positions 2823–2846 (23 bp), 2869–2905 (36 bp) and at positions 2936 to 2937 (2 bp). CitMule_3 and CitMule_ARR show dissimilar TSDs (Table 1). Three out the 4 CitMule copies identified were flanked by perfect 9 bp target side duplications (TSDs) as usual for Mutator-like elements. In “cut and paste” transposition, the TE is inserted in a unique site generating perfect TSDs through scattered cleavage [9]. Table 1 and Additional file 4: Figure S1, however, show that the transposon inserted in ARR (CitMule_ARR) as well as the CitMule_3 insertion both were flanked by dissimilar sequences. The occurrence of these dissimilar sequences indicates that each TE end was inserted in a different target site. These TSDs with dissimilar sequences are referred in this work as “dissimilar” TSDs.

**Table 1:** **Mutator-like elements of the CitMule family**

**Element**	**Chr.**	**Frame**	**Pos.**	**Size (bp)**	**SNV 22** ^**a**^	**SNV 292** ^**a**^	**SNV 313** ^**a**^	**SNV 6757** ^**a**^	**SNV 6895** ^**a**^	**SNV6926** ^**a**^	**5′ TSD** ^**b**^	**3′ TSD** ^**b**^
CitMule_1	3	+	6785326	6926	G	G	G	C	A	T	TTGTTGAAA	TTGTTGAAA
CitMule_2	4	+	25089221	6804	G	A	A	T	G	C	TTTTCGGTT	TTTTCGGTT
CitMule_3	4	-	25630357	6926	G	A	A	C	A	T	AACCGAAAA	AAAAATAAA
CitMule_4	4	-	14449845	6863	A	G	G	C	G	C	TTATTTTAA	TTATTTTAA
CitMule_ARR	3	-	6785296	6926	G	G	G	C	A	T	TTTTGAATT	TATGGTCTT

^a^Discriminatory SNVs between different CitMules.

^b^Target site duplication sequences.

ORF prediction performed with Genscan [24] with the 4 complete elements showed that the putative transposable elements contained a 5 exons transcript and that the largest exon 1 was conserved in the 4 elements (Figure 3). The ORFs of CitMule1 and _2 coded for proteins 795 aa long, while CitMule_3 and _4 produced proteins of 805 aa. Motif analysis with InterProScan [25] revealed that the predicted proteins contained a MULE transposase domain as usually found in the Mutator superfamily of transposable elements [11,12,26], in addition to a FAR1 DNA binding domain and a SWIM-type Zinc finger motif (Additional file 5: Table S4).

A MEGABLASTN search performed against the citrus EST of the GenBank yielded a total of 7 ESTs unequivocally derived from CitMule_1 and _2 and another 17 ones identical to CitMule_3 and _4 fragments (Additional file 6: Table S5). Furthermore, the presence of transcripts from the different CitMUles was unequivocally confirmed through RNA-seq analyses in C*. clementina* and another additional 11 species from mayor citrus groups including mandarins, oranges, lemons, pummelos and citrons (to be published elsewhere). Therefore, the data indicated that these elements were transcriptionally active.

The predicted protein sequence of the CitMule elements was aligned against those from other Mutator-like elements previously described in other species: MoSB-1(AAD27572) from *Sorghum bicolor*, MoOS-521 (BAA92521), and Os3378 (AP008211) from *Oryza sativa*, Jittery (AAF66982), TRAP (CAB51950), TED (AGR45850), and Mudr (AAA21566) from *Zea mays*, and Far1 (AAD51282), AtMu1 (AAG52094) and AtMu6 (AAD19776) from *Arabidopsis thaliana* (Additional file 7: Figure S2). The phylogenetic analysis performed with the Neighbor-Joining method [27] indicated that CitMule elements were clearly related to Jittery, a Mutator-like element from maize [28]. In order to identify additional citrus Mutator-like elements in the CLE genome a BLASTN search was performed using the exon 1 sequence as a query. A total of 143 sequences longer than 500 bp and with significant similarity were obtained. These sequences analyzed by the Neighbor-Joining method [27] produced the phylogenetic tree shown in Additional file 8: Figure S3. Based on these results the citrus MULEs can be grouped into 6 subfamilies, named Mutator-Like I to VI. CitMule elements clustered in subfamily I. It is worth to mention that the phylogenetic relationships between CitMule_1, 2, 3 and 4 showed in Additional file 7: Figures S2 (protein sequences) and Additional file 8: FigureS3 (genomic sequences) were slightly different probably due to the inclusion of intron sequences in the analyses of Additional file 8: Figure S3. Mapping of the MULE sequences on CLE chromosomes showed that the 6 subfamilies were interspersed randomly in the genome (Additional file 9: Figure S4).

### Insights into the CitMule 5′end

**Figure 4:**
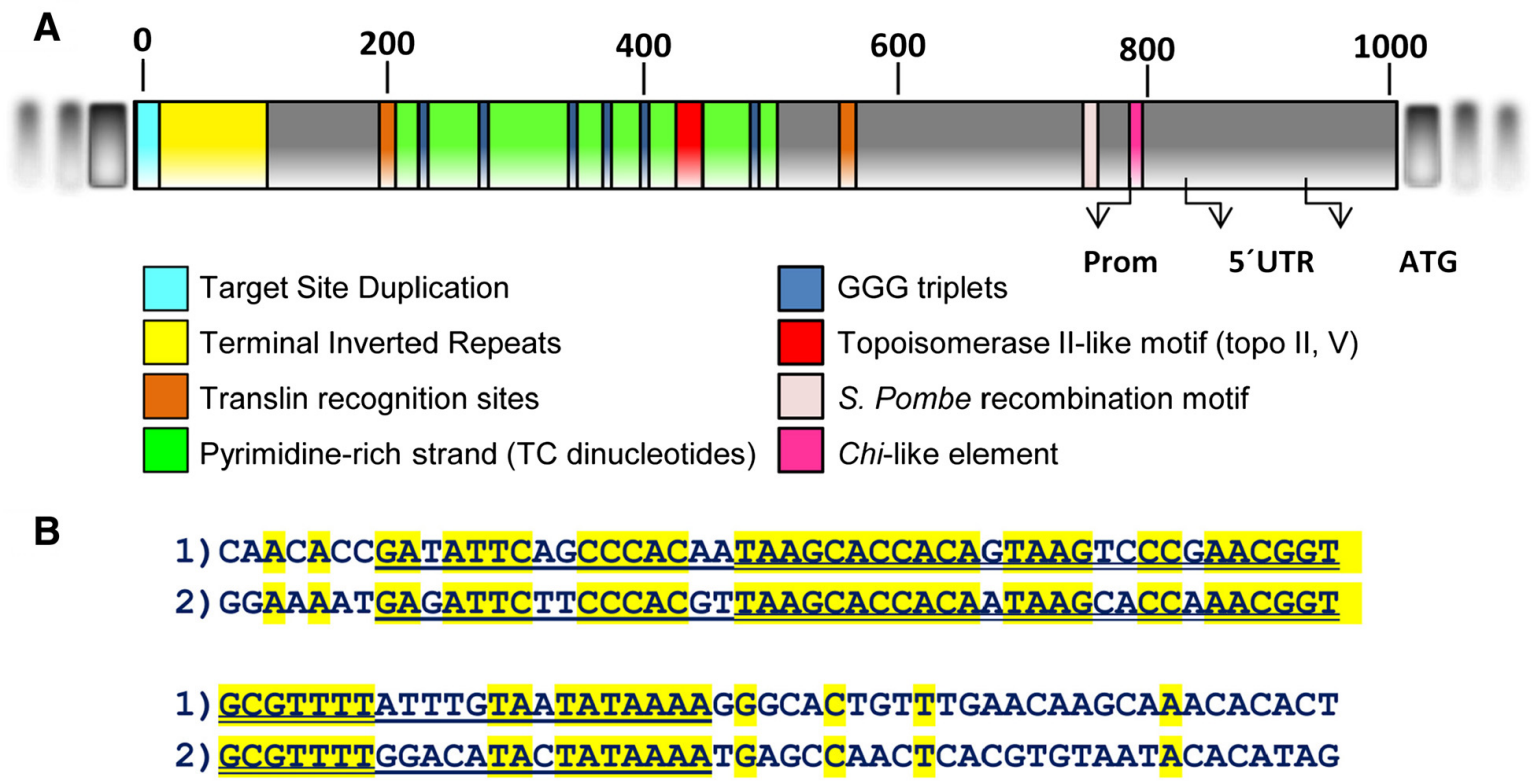
**Conserved motifs in the Cit_Mule_1 terminal region. A)** Schematic representation of the terminal 5′ sequence of Cit-Mule_1 showing the TSD (light blue), a fragment with the terminal inverted repeats (yellow), two translin recognition sites (brown), TC stretches (green), GGG triplets (dark blue), a putative topoisomerase II-like motif (red) and two specific DNA motifs of recombination hot spots identified in *Schizosaccharomyces pombe* (pink) and *Escherichia coli* (magenta). Arrows show the first nucleotide of the promoter, the first one of the 5′UTR and the first ATG of the protein according to the ORF prediction. **B)** Sequences of the 100 terminal-most nucleotides of CitMule_1. The “1” sequence represents the upstream terminal end of CitMule_1 and the “2” sequence the downstream end. The double-underlined sequences indicate tracks with 88% similarity, while the underlined sequences denote tracks with lower similarity (78%).

The terminal 5′ end of the CitMule element included several putative sequence motifs (Figure 4A) previously implicated in recombination in other organisms [21,29-31]. Thus, in this terminal end there were two translin recognition sites flanking an obvious TC rich stretch containing 6 GGG triplets, and a topoisomerase II-like motif. Other relevant sequences such as two specific recombination hot spots identified in the fission yeast *Schizosaccharomyces pombe* and the bacterium *E. coli* were also detected upstream to the pyrimidine rich stretch. Some of these motifs or elements have been found to be over-represented in the vicinity of gross deletion breakpoints in human inherited disease and cancer [2]. In this comprehensive study, the authors reported that polypyrimidine tracts as well as a number of recombination-associated motifs, such as translin binding sites and the bacterial Chi-like element were certainly over-represented at translocation breakpoints.

The most conspicuous element of this list is the pyrimidine/purine rich track (TC/GA)n. In humans, the presence of TC stretches in sites of sister chromatid exchange [32] and at the breakpoints of hybrid genes [33] has been known for a long time. The pyrimidine rich tracks are structures that, like purine pyrimidine mirror repeat sequences and palindromes of polypurine/polypyrimidine DNA stretches (see below), can readily form triplexes adopting the triple helical H-form of DNA [30,34]. Since H-DNA is partially single-stranded it may be susceptible to nuclease attack that could then facilitate recombination. Moreover, the TC rich track of CitMule also contains 6 GGG triplets that eventually might have the possibility of forming quadruplex DNA, a highly stable structure derived from double stranded GC-rich sequences [35]. Although unequivocal evidence for a specific role on chromosomal translocations in humans is still lacking, triplex DNA and G-quadruplex have been clearly implicated in genomic instability [30].

Translin is a multimeric protein that recognizes the single-stranded ends for DNA repair and potential translin binding sites including those detected in CitMule (GC[T/C]CTG [C/T]T) have been found at translocation breakpoints in many cancer diseases [36]. Chi-like elements, as the one found in CitMule (GCTGGT), are mediators of prokaryotic recombination and have been extensively reported in association with oncogenic translocation and gross deletions breakpoints in humans. It has been suggested that the Chi-like sequence elements may represent a class of recognition element for recombinases [2]. In humans, a plethora of papers have reported that topoisomerase consensus II cleavage sites have been observed in the vicinity of translocation breakpoints at a diversity of genes and in several inherited disease-associated deletion breakpoints [29,33]. The sequence detected in CitMule (CTCATCTCGCTGCTCTCT) exhibits an 89% identity with the human topoisomerase II recognition site (topo II, v) [36]. In addition to all these elements found in humans, this CitMule terminus also contained another short motif (CCAATCA) that has been significantly associated with DSBs hotspots in the genome of *Schizosaccharomyces pombe* [31]. In spite of the fact that these motifs in general are either relatively short or highly redundant and, therefore, their chance occurrence at breakpoint junctions may be simply accidental, it is rather striking that all these elements accumulate exclusively in the 5′ terminus of the CitMule. This singularity is exemplified, for instance, in Additional file 10: Figure S5 that shows that the number of TC dinucleotides, in a 100 bp basis, in the TC rich track of this terminus was 6 or 7-fold higher that the average of TC repeats detected in the 800 bp fragments flanking the TC track. Thus, the accumulation of several universal recombination motifs in this end appears to indicate that they play a role in the transposition mechanism of the CitMule element.

Another striking observation regarding the structure of CitMule is that this element does not contain long (100–200 bp) terminal inverted repeats (LTIRs) structures with high similarity (around 95%), as usual in most TIR-MULEs and Mu elements in many plants including *Arabidopsis* [10]. As shown in Figure 4B, CitMule 1 exhibits shorter degenerated TIR motifs showing much lower sequence similarity (17 bp 100%; 34 bp 88%; 65 bp 78%) and therefore appears to belong to a non-TIR-MULEs group according to the definition in Yu et al. [10]. The biological significance of these degenerate sequence motifs within the subterminal regions remains unknown although it has been suggested that they, as the longer TIRS, also may correspond to transposase recognition sequences or to cis-factors for transposase binding.

**Figure 5:**
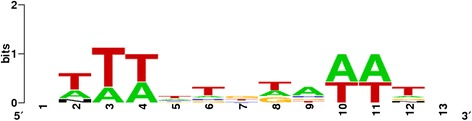
**Consensus sequence of insertion sites of CitMule elements.** Consensus sequence of insertion sites of CitMule elements. Consensus sequences were obtained with the Weblogo analysis (Crooks et al.2004) using the 7 available target site sequences including the three different 9 bp TSDs of CitMule_1, CitMule_2 and CitMule_4, and the 4 different 9 bp “dissimilar” TDs of CitMule 3 and CitMule_ARR (i.e. the 2 different sequences flanking CitMule 3 and the 2 different sequences flanking CitMule_ARR). For the consensus analysis, the immediate 13 positions upstream and downstream of CitMule termini were examined. The preferred insertion site of CitMule are the bendable AT-repeat triplets represented in the positions 2 to 4 and 10 to 12 and the precise insertion takes place between the positions 2 and 3 (or 10 and 11) in one strand and 10 and 11 (or 2 and 3) in the other. The y-axis represents the strength of the information.

On the other hand, the sequences of the 9 bp TSDs of CitMule_1, 2 and 4, as well as those flanking the insertions of CitMule_3 and CitMule_ARR, (“dissimilar” TSDs) provided a consensus sequence of the CitMule transposase insertion site (Table 1 and Additional file 4: Figure S1). While TSDs and “dissimilar” TSDs flanking the CitMule elements are certainly TA rich sequences, Figure 5 suggests that the preferred insertion sites of CitMule are bendable A/T triplets. The precise transposon cleavage in each strand takes place between the first and second nucleotides (5′ to 3′) of these A/T triplets, so that two out of the three nucleotides are necessarily included in each end of the TSD. Except the central base of the TSDs, the other 4 bases at the middle of the TSD also are preferentially A/T rich.

### Allele frequency analysis: identification of chimerism and a meiotic recombination motif

**Figure 6:**
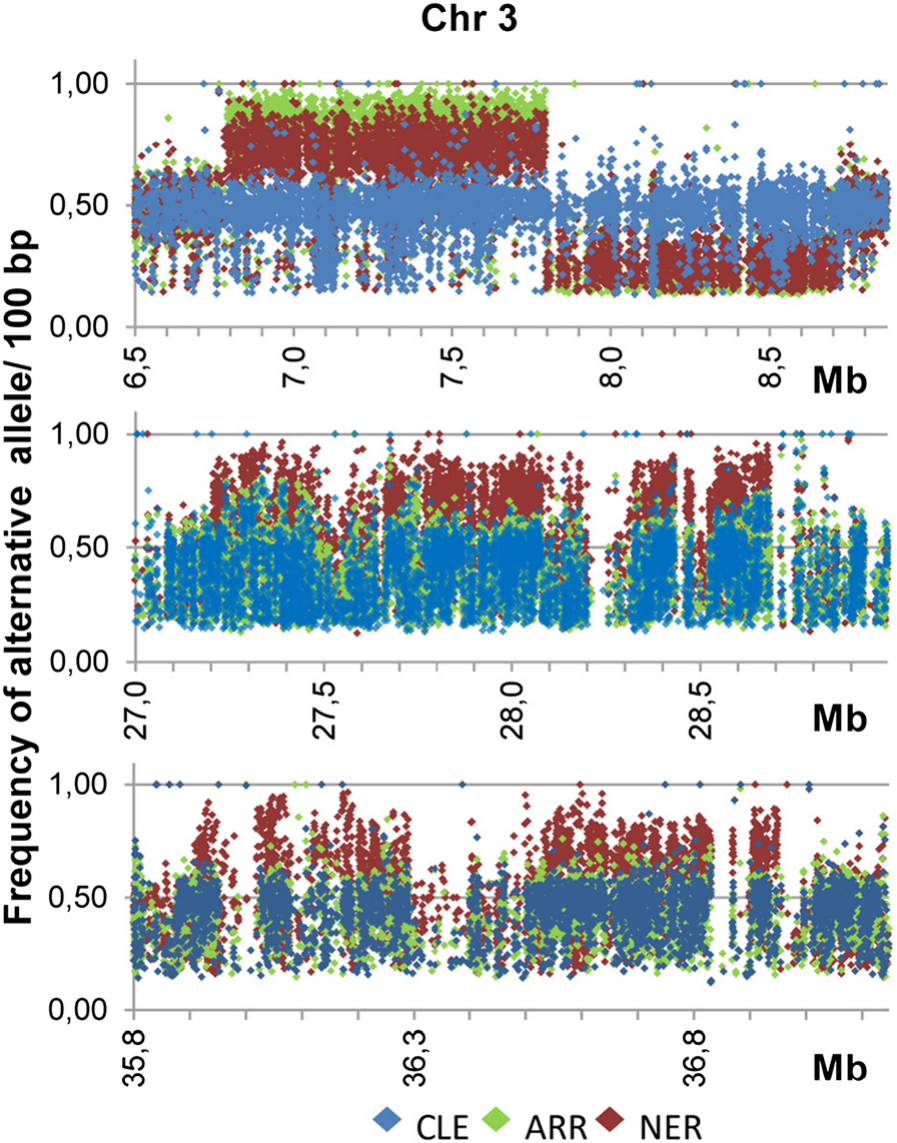
**Frequency of the alternative allele on chromosome 3.** Frequency of alternative allele in 100 bp windows in the 3 stretches of chromosome 3 corresponding to the deletions identified in ARR and NER. Frequency was calculated as the number of reads of the alternative allele with respect the total number of reads. The partner of shift detected in the allelic frequency of the 6, 7–8, 6 Mb deletion in both ARR and NER indicates the occurrence of a meiotic recombination event in the genome of reference (www.phytozome.net).

Previous haplotype analyses in the citrus reference genome indicated the presence of a crossover at the beginning of chromosome 3 [37]. In the current work, the analyses of allele frequency along the deletions detected in chromosome 3 shown in Figure 6 as well as the average calculations of these frequencies presented in Additional file 11: Table S6, clearly pointed to a chimeric nature of the ARR and NER genotypes. As explained below, chimerism allowed the precise location of the crossover and the identification of the associated DNA motif. The calculations in Additional file 11: Table S6, based on illumine reads including all positions detected in these stretches, indicated that in the deletion common to both mutants the reference allele was still present (20–30%), i.e. heterozygous SNVs were not transformed to homozygous SNVs in spite of the hemizygous deletion that removed the reference allele. (Since the crossover took place in the genome of reference but not in CLE, ARR or NER, it is convenient to mention that in this Table and in the following information concerning allelic frequency, it was assumed that the whole 6,7-8,6 Mb deletion had lower allelic frequency for the reference allele and in this way is presented the information). Chimerism was further ascertained through sequencing of PCR products after direct amplification (Additional file 12: Figure S6) or after TA cloning of stretches of the deletion (Additional file 11: Table S6). The PCR data showed normal heterozygous SNVs in CLE and reduced representation of the reference allele in vegetative and reproductive tissues of both mutants. Plant chimerism is not unusual since shoot organogenesis has a multicellular origin [38] and the presence of ARR and NER chimeras may easily have arisen after somatic mutation during the first cellular divisions of the original cells.

Moreover, Figure 6 reporting the frequency of alternative allele in the deletions identified in ARR and NER shows a sudden shift in the allelic frequency detected in the 6,7-8,6 Mb deletion in both mutants. This shift indicates the occurrence of a meiotic recombination event in the reference citrus genome [37]. According to the allelic frequency shift the recombination hotspot was located in a 260 bp track delimited by position 7797493, corresponding to the last SNV in the mutants with prevalence of the alternative allele, and position 7797753 corresponding to the first SNV with prevalence of the reference allele (Additional file 13: Figure S7). It is worth to mention that these SNV were evident because of the chimeric nature of the mutants. Although the proportion of bases was exactly the same in the 260 bp sequence included between these 2 markers and in the surrounding sequences, the number of TTC triplets in a 260 bp basis, progressively increased from 1 to 11, from position 7796194 to the track containing the hotspot. In this track, there was a TTC-trinucleotide repeat composed of 7 triplets starting at position 7797537. This sequence is very similar, if not the same, to the recently CTT-repeat motif identified in *Arabidopsis* as being enriched at meiotic crossover hot spots in *Arabidopsis* [20,21]. This observation is relevant because long GAA/TTC tracks, that cause Friedrich’s ataxia [39], for example, elicit profound mutagenic, genetic instability and major recombination behaviors. In yeast, these trinucleotide repeats strongly stimulated mitotic crossovers and were preferred sites for chromosome breakage [40], double-stranded DNA breaks and terminal deletions. Stimulation of plasmid-plasmid recombination has also been observed for GAA/TTC repeats in *E. coli*, [41]. In these recombination studies there was a positive correlation between the length of the shorter tracts and the frequency of recombination because the process was significantly hampered by the ability of longer tracks to form “sticky DNA”. Namely, short GAA/TTC repeats (no longer than 30 units) that can exist in the cell as a B-DNA duplex or a triplex, but cannot form the sticky DNA structure due to their length, are excellent substrates for intramolecular recombination [41]. The authors of this work also concluded that triplex structures formed by short tracts were responsible for the recombination hot spot activity of GAA/TTC repeats. Therefore we propose that the GAA/TTC track identified at positions 7797537–7797558 is the sequence responsible of the hot spot activity that resulted in the crossover identified in the citrus reference genome.

### The TTC-repeat motif produces genome instability at different levels

The idea that recombination motifs are drivers of genome instability is not new. Thus, in addition to the specific role on meiotic crossovers, a number of recent reports also suggest that meiotic recombination motifs are similarly associated with chromosomal rearrangements. In humans, a common sequence motif found in hypervariable minisatellites and clustered in the breakpoint regions of both diseases and mitochondrial deletion hot spots was clearly implicated in genome instability [42]. Furthermore, other human hotspot sequence motifs and repeat elements also showed an interesting connection between meiotic recombination and genes with disease associated chromosomal rearrangements [43]. It is now widely accepted that some tracks of genomic DNA that adopt non-canonical B-DNA structures like DNA-hairpin, cruciform, Z-DNA, triplex and tetraplex are represented as hotspots of chromosomal breaks, homologous recombination and gross chromosomal rearrangements [44]. Intra-molecular triplex, for instance, are overrepresented in the human genome and generally found near promoter regions and recombination hotspots. The TTC-repeat in particular has been extensively studied because expansions of these tracks are associated with the human disease Friedrich’s ataxia [39]. These triplets are prone to form DNA triplexes, the major components of H-DNAs, unusual DNA structures formed in homopurine-homopyridine regions of supercoiled DNA [45,46]. The H form consists of an intramolecular triple helix formed by the pyrimidine strand and half of the purine strand, leaving the other half of the purine strand single stranded. The existence of single stranded purine stretches and the hyperreactivity of this conformation to S1 nuclease [46] appear to be the reason of the strong association of the TTC- and other repeat motifs to recombination and also to the generation of chromosomal rearrangements. Furthermore, it has been demonstrated the occurrence of replication-associated intramolecular junctions between TTC-repeats and other homopurine-homopyrimidine tracts [47]. These unusual molecular junctions are accompanied with breakage that appears to be physically linked to non-GAA DNA sequences and could result from exposure of the single stranded DNA. In this configuration, chromosome fragility takes place in proximity to GAA repeats while in the vast majority of reported rearrangements the motifs are rather coincident with the breakpoints of the genomic alterations [40,42-44]. However, there is a clear-cut difference between the involvement of TTC-repeats in the gross genomic chromosomal rearrangements reported to date and the deletions observed in ARR an NER, since in these mutations the motif is centered just in the middle of the deletions (Figure 6). This difference implies that chromosome break in the mutants was not due to the TTC-motif itself, but instead the motif by a mechanistic way located in close proximity the two initially separate target sites in each mutation. Since the formation of triplex DNA implies a sharp bend in the DNA molecule, these observations predict a functional model that is consistent with a duplex-strand separation mediated by the TCC-repeat motif that does not end in recombination or chromosome break but fold back the DNA initiating a loop configuration that brings together both targets. Therefore, the TTC-motif may act as driver of genomic instability at different levels since in Clemenules there was no indication of TTC activity, while in the citrus reference genome, this motif provoked a crossover, in NER formed a loop and in ARR formed a loop that facilitated a TE-mediated deletion.

### Proposed model for the ARR TE-mediated deletion

**Figure 7:**
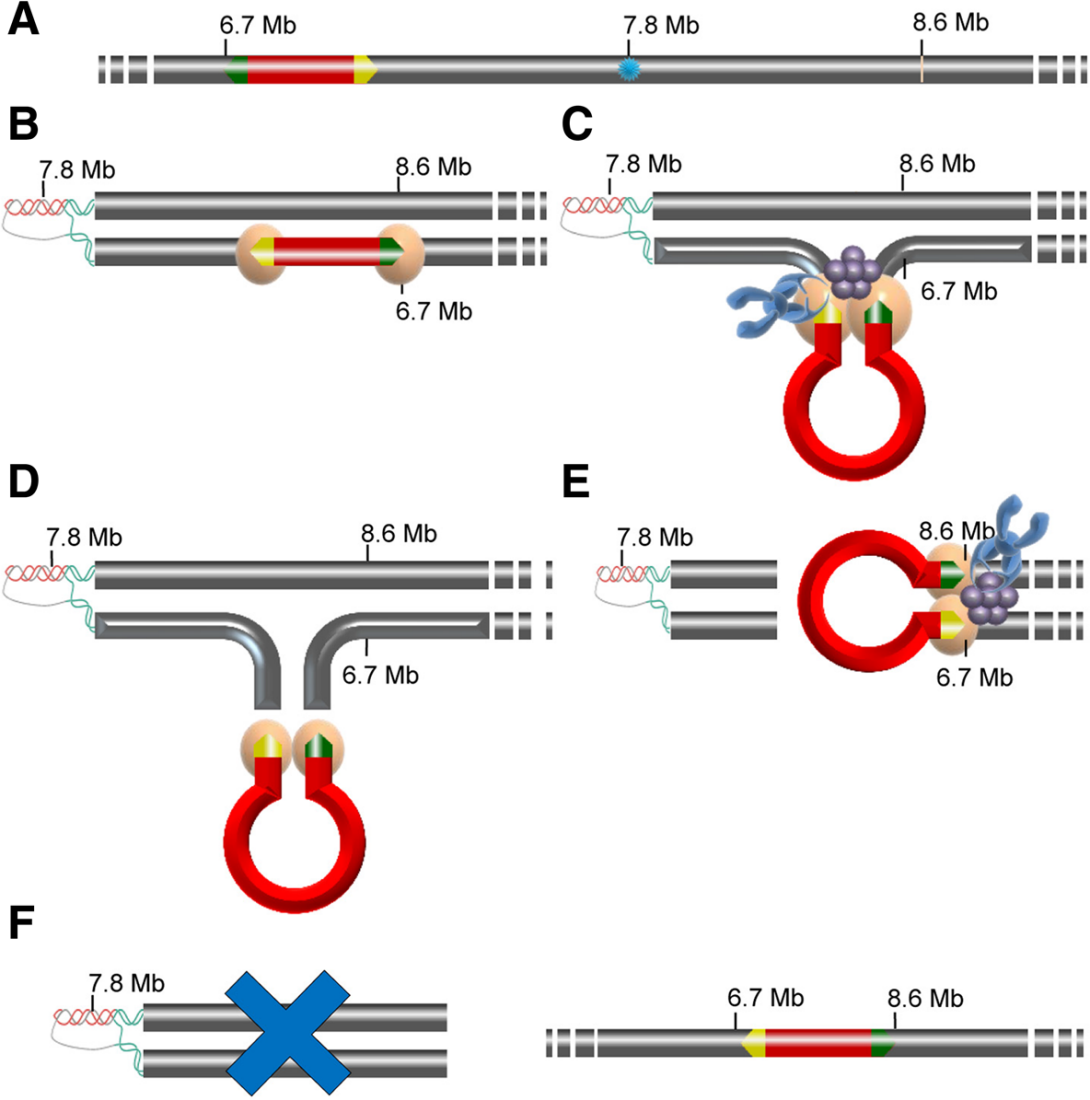
**A proposed model for the ARR TE-mediate deletion. A**. Structure of the chromosome 3 (gray bar) of the original variety, CLE, showing the occurrence of CitMule (position 6785327; red track) with the transposon inverted repeats (TIRs, green and yellow arrows) and a putative recombination hotspot (position 7797540; blue star) formed by a GAA/TTC track. **B**. A triplex DNA structure is formed in ARR at the recombination hotspot generating a loop that eventually brings the upstream end of CitMule near to the vicinity of position 8686356. Two transposase monomers bind to the transposon inverted repeats. **C**. Looping of the transposon brings the two ends of the transposable element close together. Transposase monomers form a dimer generating a paired-end complex. The complex recruits other involved proteins such as topoisomerases (blue object) and translins (purple object) resulting in the formation of a synaptic complex. Other putative trans-factors are not represented. **D**. Transposase, first, cuts the CitMule away from the flanking donor DNA and after cleavage the transposase/complex is released. **E**. In normal insertions, it is expected that transposase encounters a unique target side for insertion, generating the same TSD sequence in each side of the insertion. In ARR, the transposase/complex recognized two different CitMule target sites separated by approximately 2 Mb. One of these was the target site at position 8686356 and the other one was at position 6785296. Note that this position and the original position of CitMule are separated by 35 bp. **F**. Transposase catalyzes the inverted insertion of the transposon between these 2 target sites provoking in this way the release of the fragment spanning from one target site to the other one. Therefore, these events resulted in both an inverted insertion of CitMule and a 2 MB deletion in chromosome 3 as observed in ARR.

Based on the information reported above, Figure 7 summarizes a proposed model for the deletion mechanism that occurred in the original CLE variety and generated ARR, the derived variety. Data in Table 1 indicates that CitMule_1 was the element that was excised from its original position at 6785327 bp in chromosome 3 (Figure 2A) and thereafter was inserted in inverted orientation in the same chromosome 3 but at position 8686356 bp. The model assumes that a triplex DNA structure was formed at the GAA/TTC recombination hotspot at position 7797537–58 [30,34], generating a loop that brought the upstream end of CitMule near to the vicinity of a CitMule insertion target site at position 8686356. According to current knowledge, most DNA transposons mobilize by a ’cut and paste’ mechanism with a central role for transposase that binds at TIRs, excises the transposon from its existing genomic location and pastes it into a new genomic location [48]. The observations presented above suggest that after transposase binding to the transposon inverted repeats, a complex may be formed with the possible participation of topoisomerase and translin and the bacterial Chi and/or the *S. pombe* trans-elements. In normal insertions, it is expected that transposase encounters a unique target site for insertion. Since the cleavages of the two strands at the target site are staggered, this results in a duplication of the target-site, i.e. the same sequence in each side of the insertion. In ARR, the transposase/ complex recognized two different CitMule target sites in the same chromosome 3. One of these was the target site (position 8686356) that after lopping was physically located near to the upstream end of CitMule and the other one was at position 678529, both separated by approximately 2 Mb. Transposase catalyzed the inverted insertion of the transposon between these 2 target sites provoking in this way the release of the fragment spanning from one target site to the other one. As observed in ARR, these events resulted in an inverted insertion of CitMule, a pair of dissimilar TSDs flanking the transposon and a 2 MB deletion in chromosome 3.

### Chromosomal rearrangements in irradiated NER

Several genomic lesions, i.e. three deletions in chromosome 3 and two more in chromosome 8 plus a translocation from chromosome 8 to 6 were identified in NER, the variety generated through fast neutron irradiation (Figures 1B and 2B). It is believed that most genomic lesions induced in cells exposed to ionizing radiations are caused either directly by DNA ionization or indirectly by free radicals. Ionizing radiation frequently causes “clustered DNA damage” when the ionization track induces several lesions in the DNA within a couple of turns [7,8]. Furthermore, it is generally accepted that DSBs in the DNA is the main cause of these lesions since misrepair or lack of repair of DSBs apparently triggers most mutations and chromosomal rearrangements found in irradiated tissues. Sankaranarayanan and Wassom [6] have proposed that the mechanisms involved in the repair of radiation induced DSBs in mammalian cells are likely the same that those naturally occurring, and have hypothesized that incorrect DSB rejoining of broken ends in the same chromosome may generate an interstitial deletion. While no attempts have been made in the current work to test this hypothesis, it is intriguing that the breakpoints of the 2 Mb deletion detected in chromosome 3 of NER, (positions 6782589–8724143) are just equidistant to the repeat motif location (position 7797537), as in ARR. This observation is also compatible with the formation in NER of a triplex DNA configuration promoted by the TTC motif and, therefore, it is coherent to suggest that the same ionization track provoked both breakpoints generating “clustered DNA damage” [7,8] in the two initially distant sites. Since the boundaries of the other 2 deletions in chromosome 3 could not be exactly defined, the presence of these repeats around the center of the deletions could not be precisely ascertained, but the fragment involved in the rearrangement detected in chromosome 8 of NER (positions 12602311–13635996) also contained at least three different TTC-repeat motifs roughly located around the midpoint of the rearrangement.

### Gene content in the ARR/NER deletion

Genes present in the ARR and NER deletion were obtained from the annotation of the clementine genome available at http://www.citrusgenomedb.org/. A total of 244 primary transcripts were predicted in this region. Functional annotation of the genes was carried out with BLAST2GO [49] (Additional file 14: Table S7). In the listing of annotated genes located at the deletion there were several transcription factors and genes both related to hormone responses and associated with early ripening, the main characteristic of the ARR and NER phenotype (Additional file 1: Table S1). Among them, there were genes related to chlorophyll and carotenoid biosynthesis, carbohydrate and sugar metabolic processes and also to acid metabolism (Additional file 14: Table S7). This region also contained 4 different protein-COBRA like genes that presumably encode a plant-specific glycosylphosphatidylinositol-anchored protein [50] and three additional genes implicated in phosphatidylinositol biosynthetic processes. This observation deserves further investigation because it has been shown that COBRA overexpression in transgenic tomato promoted early fruit development and delayed senescence [51]. Similarly, a few genes apparently related to other minor ARR/NER traits such as water transport and defense response to fungus, were also detected. ARR and NER certainly differ in one pivotal characteristic because ARR is a self-incompatible variety while NER is basically a sterile variety. However, the sterility in NER should be attributed to the irradiation treatment [52] and specifically to the generation of additional deletions (Figure 1) that presumably compromised correct chromosome pairing [6] during meiosis.

With no additional information, however, the diversity of the ARR and NER phenotypic traits suggests that this phenotype is a consequence of the combined effects of many deleted genes rather than a particular effect of one single or a few genes. The concept that deletions are more likely to manifest themselves as combination of multiple developmental alterations have previously been settled in other fields such as the irradiation induced deletions in human germ cells [6].

## Conclusions

Aside from the identification of Mules in citrus and the first report on the conspicuous accumulation of several motifs involved in human chromosome breaks in a TE end terminus, this work highlights overall two major findings: the identification of a TTC-repeat as a motif physically responsible of meiotic recombination in plants and its involvement in the generation of gross deletions. The existence of gross deletions implies the presence of double-stranded breaks in two initially distinct locations that became physically located in close proximity and are rejoined by illegitimate repair. However, there is not a reasonable explanation of how two genomically separate sites become located in close proximity. We show compelling evidence in two different genotypes (a spontaneous and irradiate mutant) consistent with the proposal that the recombination TTC-repeat motif form a triplex DNA structure generating a loop that enables the proximity of distant sequences, facilitating double strand break by Mule reaction or clustered DNA damage provoked by a ionizing track. This proposal offers a new insight in “cut and paste” transposition since it requires the involvement of a unique transposon for the formation of gross deletions and offers a simple explanation to the unresolved question of how two genomically distinct sites become located in close proximity during chromosomal rearrangements. These observations confer an active and specific double role to the identified TTC-repeat motif in fundamental processes as meiotic recombination and chromosomal rearrangements in plants.

## Methods

### Plant material and DNA extraction

Clementine (*Citrus clementina*) cultivars of Clemenules (CLE), Arrufatina (ARR) and Nero (NER) were used in this study. Clemenules is a self-incompatible genotype that is vegetatively propagated while ARR and NER are both derived from somatic CLE mutations. ARR is a bud sport and NER an induced mutant obtained by fast neutron irradiation. Genomic DNA for genome sequencing was extracted exclusively from fresh young leaves after nuclear isolation. Leafs were grinded, nuclear buffer was added and samples homogenized and filtered on Miracloth layers. Extracts were centrifuged twice and the pellet re-suspended in floating buffer and centrifuged again. Nuclei, recovered by pipetting, were homogenized, re-suspended in nuclear buffer and centrifuged. The supernatant was discarded, RNase and protein Kinase A were added to the pellet and the extract incubated at 50°C with gentle shaking and centrifuged. Nuclei in the supernatant were transferred and DNA extraction was performed by mixing the solution with an equal volume of phenol/chloroform/isoamyl alcohol (25:24:1). A second extraction with isopropanol was carried out and after centrifugation the DNA was recovered in the pellet. Three washes of ethanol were performed; the alcohol discarded and after drying DNA was re-dissolved in TE. For PCR analyses DNA was extracted from leaves and flavedo tissue with the DNAeasy Plant mini kit (Qiagen).

### Genome sequencing

Libraries were constructed using the Illumina TruSeq DNA Sample Prep standard protocol with some modifications. Briefly, 1 μg of high molecular weight genomic DNA was fragmented with a Covaris sonication device. Thereafter, DNA fragments were end-repaired and A-tailed. Adapters were then ligated via a 3′ thymine overhang. Finally, ligated fragments were amplified by PCR (10 cycles). Libraries insert sizes ranged from 400 to 500 bp. The library was applied to an Illumina flowcell for cluster generation. Sequencing was performed on a HiSeq2000 instrument using 100 bp paired-end reads. Primary analysis of the data included quality control on the Illumina RTA sequence analysis pipeline.

### Sequence processing, mapping and variant calling

Low quality bases from sequence tails were trimmed via a custom script. Afterwards, extremely short remaining lectures and those with low mean quality were also filtered out. PCR duplicated sequences were removed. Selected reads were aligned against the citrus clementine reference genome (v 1.0) using the Burrows-Wheeler Aligner (BWA) [53]. Raw mapped reads were filtered by mapping quality, sorted and indexed with Samtools [54]. Finally, selected reads were realigned following the GATK [22] variant detection protocol. High quality mapped reads were used to detect SNVs and Indels. These were called with the GATK software and variants were labelled according to the quality control scores provided in the tool. Finally, labelling was used to define high quality sets of variants with low false positive rates.

### PCR analyses

Gene dosage measurements were determined through Real-time quantitate PCR, on a LightCycler 2.0 instrument (Roche) using the LightCycler FastStart DNA MasterPLUS SYBR Green I kit (Roche) essentially as described in Rios et al. [55]. Each individual PCR reaction contained 2 ng of genomic DNA. Cycling protocol consisted of 10 min at 95°C for pre-incubation followed for 45 cycles of 10 s at 95°C for denaturation, 10 s at 60°C for annealing and 20 s at 72°C for extension. Specificity of the PCR reaction was assessed by the presence of a single peak in the dissociation curve after amplification and through size estimation of the amplified product. Gene dosage measurements were calculated comparing the ratio of target sequences inside and outside of the deletion in the three genotypes. PCR and normalized calculations were repeated in at least three independent samples. Non-quantitative PCR reactions contained 100 ng of genomic DNA, 0,6 μM of each primer and 0.5X of 2xPhusion master mix (Phusion High-Fidelity PCR Master, Cat. no F-532S, Thermoscientific). Cycling protocol consisted of 1 min at 98°C for pre-incubation followed by 35 cycles of 10 s at 98°C for denaturation, 20 s at 60°C for annealing and 60 s at 72°C for extension and one cycle of 10 min at 72°C for final elongation. Specificity of PCR reaction was confirmed by agarose gel electrophoresis and direct sequencing of the PCR product. For TA cloning, a 539 bp fragment corresponding to Ciclev10024595m.g locus was amplified using the Advantage HD Polymerase Mix (Clontech, Mountain View, CA, USA) with the GD1 specific oligos. Primers used are listed in Additional file 15: Table S8.

### TA cloning

TA cloning was performed following the pGEMT-Easy vector system protocol (Promega, Madison, USA) according to manufacturer’s instructions.

### Availability of supporting data

CLE Illumina reads were submitted to the NCBI Sequence Read Archive with the experiment accession number SRX371962 (http://www.ncbi.nlm.nih.gov/sra/?term=SRX371962). ARR and NER sequences were submitted to the European Nucleotide Archive with the study accession number PRJEB5808 (http://www.ebi.ac.uk/ena/data/view/PRJEB5808).

All the other supporting data are included as additional files.

## Additional files

Additional file 1: Table S1. Discriminating fruit traits of three Clementines: Clemenules (CLE), Arrufatina (ARR) and Nero (NER). Clemenules characteristics are considered to be standard for the group. Arrufatina and Nero are two mutants derived from Clemenules through spontaneous and induced mutations, respectively. Additional file 2: Table S2. Sequencing, mapping and variant calling statistics of the Illumina short read sequences of three Clementine genomes: Clemenules (CLE), Arrufatina (ARR) and Nero (NER). Total reads: after removal of duplicates; Filtered reads: after quality filter; >15×: percentage of genome with coverage higher than 15×. Additional file 3: Table S3. Rearrangements in chromosome 3, 8 and 6 of ARR and NER deduced from pair-end analyses of short read sequences with the IGV software. The table shows the start and end positions of the rearrangements and the orientation and % of pair-end reads supporting the event. The orientation of pair reads indicated that nature of the rearrangement (deletion vs. inversion or translocation), while the percentage suggested the hemizygous condition of the event. One single set of pair reads are sufficient to reveal deletions while insertions and transposition need two set of pair-ends. All rearrangements were confirmed by further PCR analyses except the deletion in chromosome 3 of NER that was ascertained by gene dosage. Note that the ARR inversion in chromosome 3 implies the occurrence of a deletion spanning from the position 6785295 to the position 8686355 and that the translocation event resulted in a 33 bp deletion on chromosome 6. Additional file 4: Figure S1. Sequences of citrus Mule insertion regions. The sequences and positions of the CitMule elements identified in CLE and ARR are shown on the two homologous chromosomes. CitMules sequences are shaded in purple, target site duplications (TSDs) in yellow, original insertion sides in orange and “dissimilar” TSDs in green. CLE is hemizygous for CitMule_1 and CitMule_4, and homozygous for CitMule_2 and CitMule_3 while ARR is hemizygous for CitMule_ARR. CitMule_3, CitMule_4 and CitMule_ARR were presented in inverted orientation. Additional file 5: Table S4. CitMule Motif analysis performed with InterProScan. Additional file 6: Table S5. Results of a MEGABLASTN search performed against the citrus ESTs of the GenBank. Additional file 7: Figure S2. Phylogenetic tree of CitMules and other Mutator like proteins from plants constructed with the neighbor-joining method. Additional file 8: Figure S3. Phylogenetic tree of the Mutator-like elements present in the CLE genome constructed with the neighbor-joining method. Additional file 9: Figure S4. Mapping of MULE sequences on CLE chromosomes. The 6 subfamilies were interspersed randomly in the genome. Additional file 10: Figure S5. Number of dinucleotides per 100 bp, in the 2000 bp sequence flanking the 5′ end of the CitMul element (arrow). Additional file 11: Table S6. Alternative allele frequency (± St dev) in the deletion identified in chromosome 3 of ARR and NER. Average allelic frequency based on Illumina reads (n > 10.000) and on PCR product sequencing (n = 16) after either direct amplification or after TA cloning are presented. Additional file 12: Figure S6. Sequencing chromatograms of PCR products amplified from regions of the ARR and NER hemizygous deletion in chromosome 3. Amplifications were performed with GD1 primers. Arrows show three heterozygous positions in CLE, the original variety, that are not converted to homozygous positions in ARR and NER. Additional file 13: Figure S7. Sequence of the haploid CLE genome, the citrus reference genome (GenBank: AMZM00000000.1), delimiting a recombination motif detected inside the common deletion in chromosome 3 of ARR and NER. The position in red at 7797493 indicates the last single nucleotide variation (SNV) position in the mutants with prevalence of the alternative allele. The position at 7797753 marks the first SNV position with prevalence of the reference allele. The green stretch starting at position 7797537, between both red markers, indicates a putative recombination motif formed by 7 consecutive TTC trinucleotides. Additional file 14: Table S7. Listing of candidate genes tentatively associated with the observed ARR and NER phenotypic traits. Additional file 15: Table S8. Specific primers for the determinations of gene dosage, allelic frequency (GD1) and chromosome rearrangement boundaries. Orientation: (+ forward, − reverse); start: position in reference genome. GD was used as control in gene dosage measurements. The transposon inversion found in Arrufatina was studied with ARR-5′ + CitMul_1 and ARR-CitMul_1 + 3′ primers. In Nero, NER-Del8-A, NER-Del8-B, NER-5′-T(8;6) and NER-3′-T(8;6) primers were used for the determination of deletion and transposition boundaries.
