# Supplementary material for: Involvement of a citrus meiotic recombination TTC-repeat motif in the formation of gross deletions generated by ionizing radiation and MULE activation
Source: BMC Genomics. 2015 Feb 13;16(1):69. doi: 10.1186/s12864-015-1280-3 (PMC4334395; doi:10.1186/s12864-015-1280-3)
Supplement: Additional file 1: Table S1. — Discriminating fruit traits of three Clementines: Clemenules (CLE), Arrufatina (ARR) and Nero (NER). Clemenules characteristics are considered to be standard for the group. Arrufatina and Nero are two mutants derived from Clemenules through spontaneous and induced mutations, respectively. [file 12864_2015_1280_MOESM1_ESM.pdf]

Table S1. Discriminating fruit traits of three Clementines

	CLE[a]	ARR[b]	NER[b]
Ripening period	Standard	Precocious	Precocious
Acidity reduction	Dec	Nov	Nov
Sugar accumulation	Dec	Nov	Nov
Color change	Mid Nov	Late Oct	Late Oct
Presence of seeds	Self-incompatible	Self-incompatible	Sterile

[a] Clemenules, [b] Arrufatina, [c] Nero
