# Supplementary material for: Involvement of a citrus meiotic recombination TTC-repeat motif in the formation of gross deletions generated by ionizing radiation and MULE activation
Source: BMC Genomics. 2015 Feb 13;16(1):69. doi: 10.1186/s12864-015-1280-3 (PMC4334395; doi:10.1186/s12864-015-1280-3)
Supplement: Additional file 2: Table S2. — Sequencing, mapping and variant calling statistics of the Illumina short read sequences of three Clementine genomes: Clemenules (CLE), Arrufatina (ARR) and Nero (NER). Total reads: after removal of duplicates; Filtered reads: after quality filter; >15×: percentage of genome with coverage higher than 15×. [file 12864_2015_1280_MOESM2_ESM.pdf]

.

Table S2. Sequencing, mapping and variant calling statistics of the Illumina short read sequences of three Clementine genomes

Variety	Total Reads[a]	Filtered Reads[b]	Paired reads	%GC	Coverage	>15x[c]	SNVs Total	SNVs Het	SNVs Hom	Indels Total	Indels Het	Indels Hom
CLE	456194154	207805481	204655262	33.16	68.95x	83.14	1414773	1400808	13965	158995	147577	11418
ARR	227338589	131179259	129256801	32.05	43.53x	81.46	1424483	1411343	13140	157942	147311	10631
NER	211454854	118363024	116579261	32.51	39,27x	81.79	1440013	1427321	12692	157923	147565	10358

[a] Total reads: after removal of duplicates
[b] Filtered reads: after quality filter
[c] >15x: percentage of genome with coverage higher than 15x
