# Supplementary material for: Involvement of a citrus meiotic recombination TTC-repeat motif in the formation of gross deletions generated by ionizing radiation and MULE activation
Source: BMC Genomics. 2015 Feb 13;16(1):69. doi: 10.1186/s12864-015-1280-3 (PMC4334395; doi:10.1186/s12864-015-1280-3)
Supplement: Additional file 3: Table S3. — Rearrangements in chromosome 3, 8 and 6 of ARR and NER deduced from pair-end analyses of short read sequences with the IGV software. The table shows the start and end positions of the rearrangements and the orientation and % of pair-end reads supporting the event. The orientation of pair reads indicated that nature of the rearrangement (deletion vs. inversion or translocation), while the percentage suggested the hemizygous condition of the event. One single set of pair reads are sufficient to reveal deletions while insertions and transposition need two set of pair-ends. All rearrangements were confirmed by further PCR analyses except the deletion in chromosome 3 of NER that was ascertained by gene dosage. Note that the ARR inversion in chromosome 3 implies the occurrence of a deletion spanning from the position 6785295 to the position 8686355 and that the translocation event resulted in a 33 bp deletion on chromosome 6. [file 12864_2015_1280_MOESM3_ESM.pdf]

Table S3. Rearrangements in chromosome 3, 8 and 6 of ARR and NER

Variety	Rearrangement	Size (bp)	Chr	Start	End	Pair ends orientation	%[a]
ARR	Inversion	6926	3	6785295	6792253	- -	14
	Deletion	1894102	3	6785325	8686355	+ +	11
NER	Deletion	1941554	3	6782589	8724143	+ -	40
	Deletion	908471	8	12602313	13510784	+ -	53
	Deletion	69130	8	13566864	13635994	+ -	50
	Translocation	721	(8;6)[b]	13634859	21997756	- +	43
			(8;6)[b]	13635580	21997789	+ -	46

[a] Percentage of pair ends supporting rearrangement
[b] Chromosomes involved in translocation
