# Supplementary figures and images for: Involvement of a citrus meiotic recombination TTC-repeat motif in the formation of gross deletions generated by ionizing radiation and MULE activation
Source: BMC Genomics. 2015 Feb 13;16(1):69. doi: 10.1186/s12864-015-1280-3 (PMC4334395; doi:10.1186/s12864-015-1280-3)

**CitMule\_1 (Chromosome 3)**

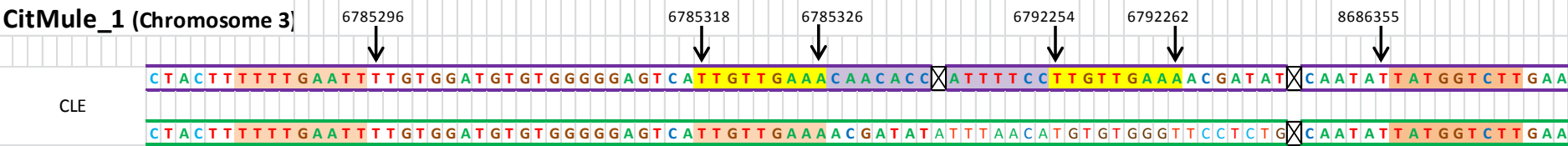

**CitMule\_2 (Chromosome 4)**

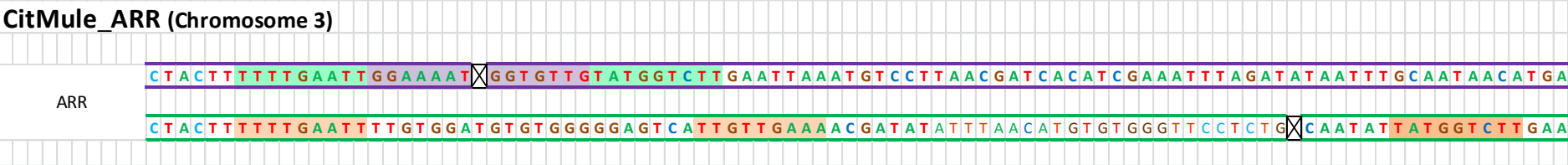

**CitMule\_3 (Chromosome 4)**

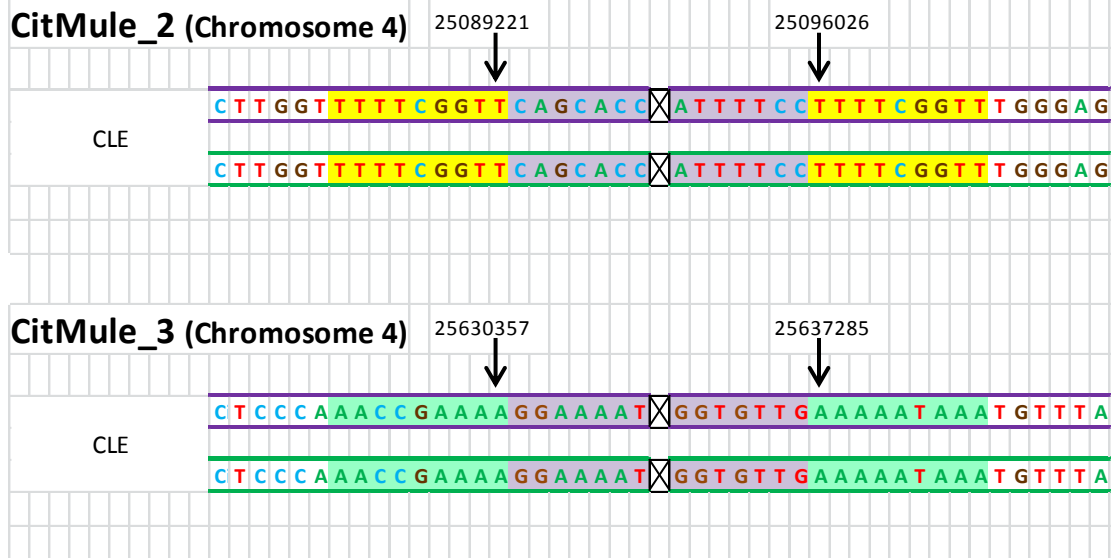

**CitMule\_4 (Chromosome 4)**

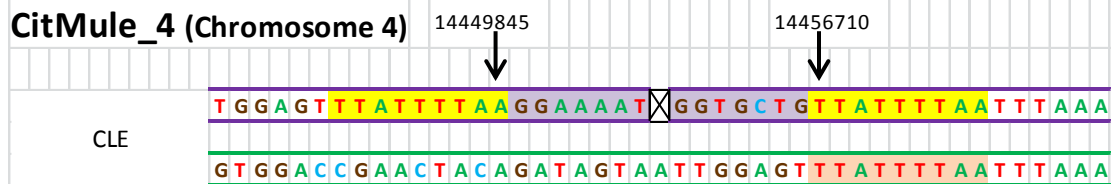

Supplement: Additional file 4: Figure S1. — Sequences of citrus Mule insertion regions. The sequences and positions of the CitMule elements identified in CLE and ARR are shown on the two homologous chromosomes. CitMules sequences are shaded in purple, target site duplications (TSDs) in yellow, original insertion sides in orange and “dissimilar” TSDs in green. CLE is hemizygous for CitMule_1 and CitMule_4, and homozygous for CitMule_2 and CitMule_3 while ARR is hemizygous for CitMule_ARR. CitMule_3, CitMule_4 and CitMule_ARR were presented in inverted orientation. [file 12864_2015_1280_MOESM4_ESM.pdf]

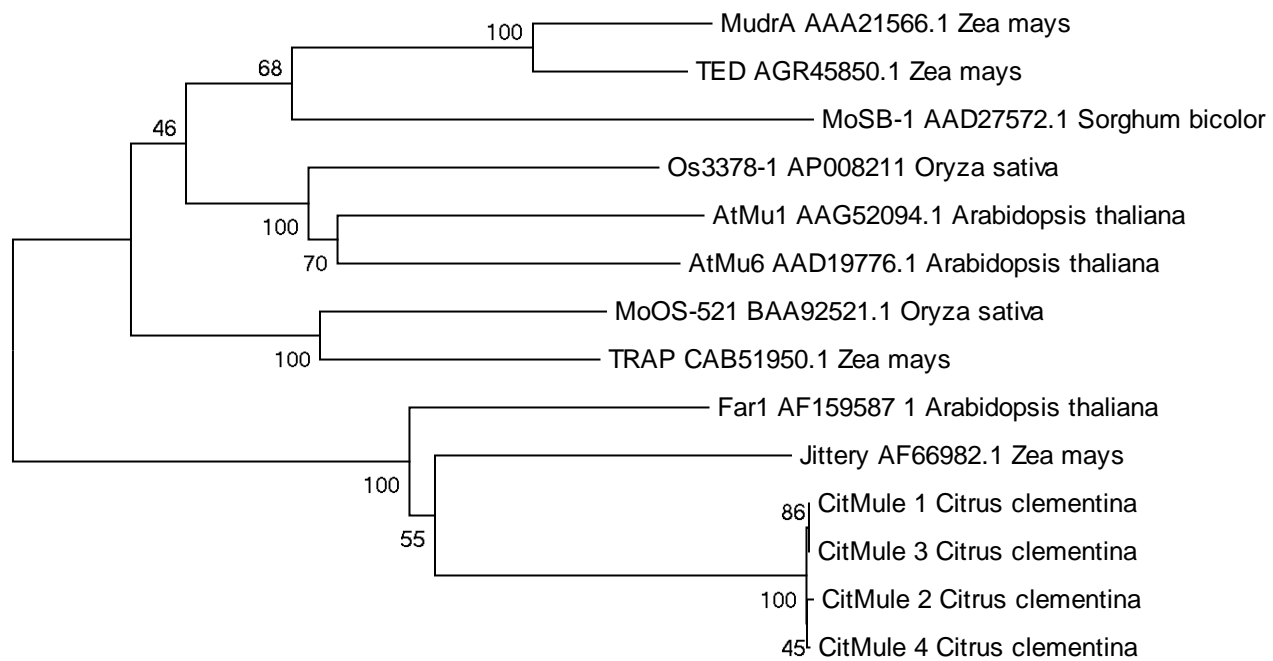

Supplement: Additional file 7: Figure S2. — Phylogenetic tree of CitMules and other Mutator like proteins from plants constructed with the neighbor-joining method. [file 12864_2015_1280_MOESM7_ESM.pdf]

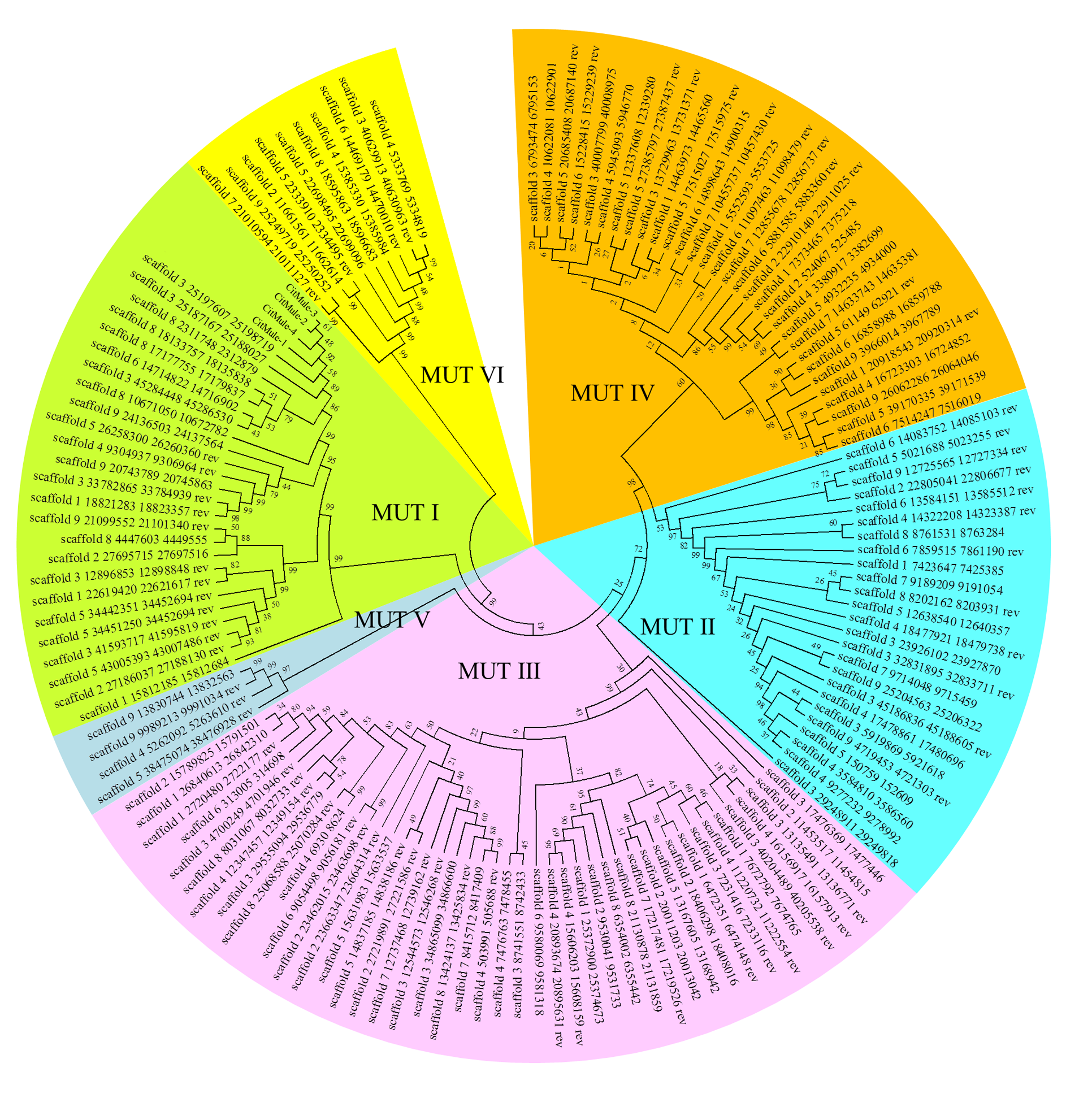

Supplement: Additional file 8: Figure S3. — Phylogenetic tree of the Mutator-like elements present in the CLE genome constructed with the neighbor-joining method. [file 12864_2015_1280_MOESM8_ESM.tif]

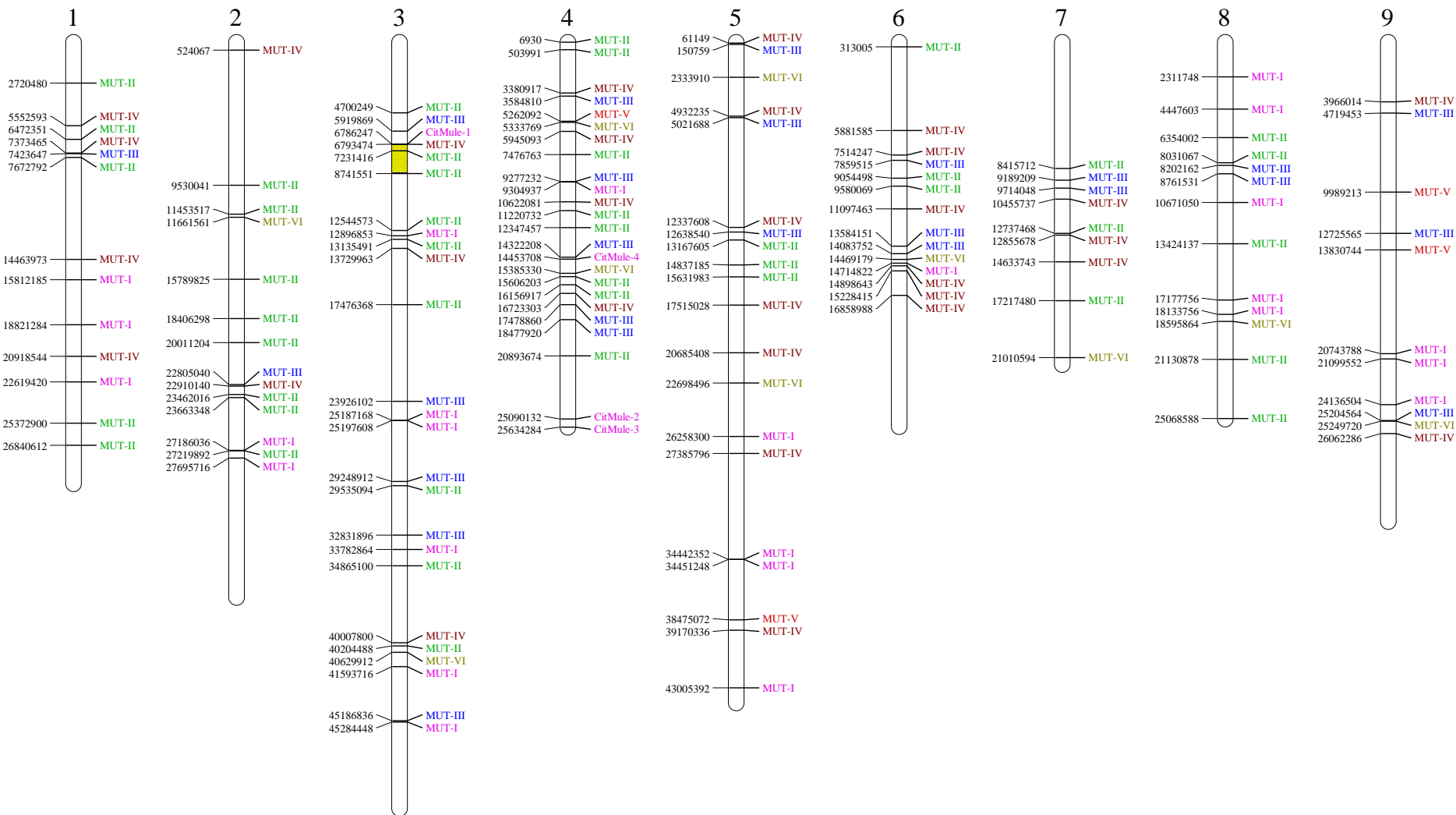

Supplement: Additional file 9: Figure S4. — Mapping of MULE sequences on CLE chromosomes. The 6 subfamilies were interspersed randomly in the genome. [file 12864_2015_1280_MOESM9_ESM.pdf]

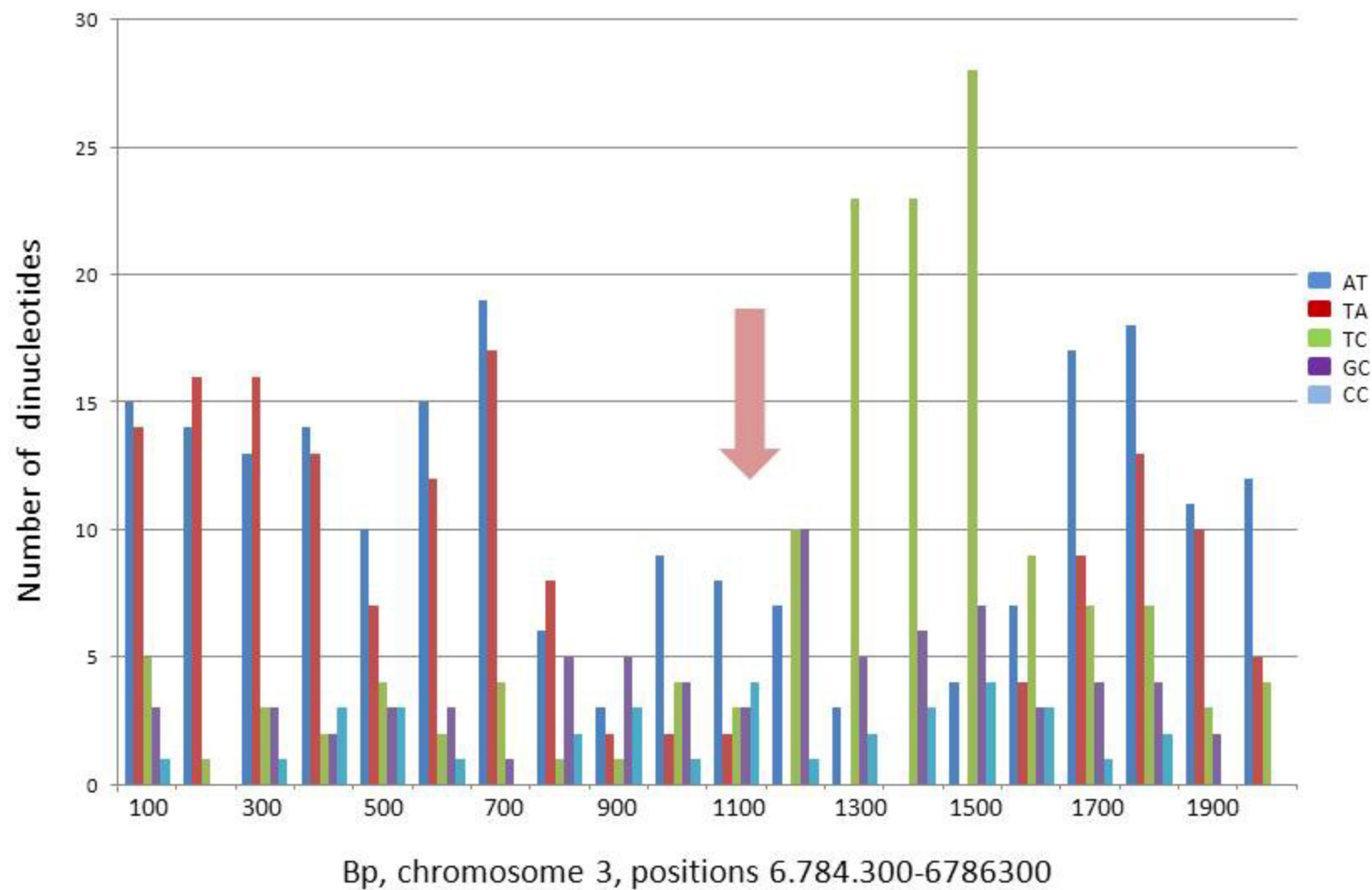

Supplement: Additional file 10: Figure S5. — Number of dinucleotides per 100 bp, in the 2000 bp sequence flanking the 5′ end of the CitMul element (arrow). [file 12864_2015_1280_MOESM10_ESM.pdf]

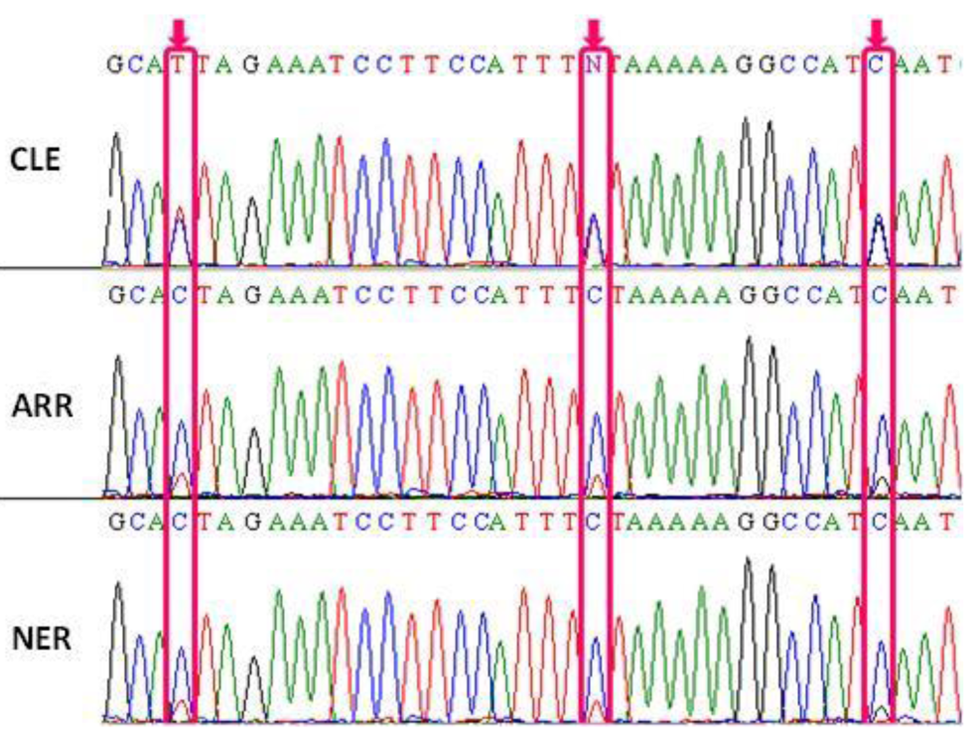

Supplement: Additional file 12: Figure S6. — Sequencing chromatograms of PCR products amplified from regions of the ARR and NER hemizygous deletion in chromosome 3. Amplifications were performed with GD1 primers. Arrows show three heterozygous positions in CLE, the original variety, that are not converted to homozygous positions in ARR and NER. [file 12864_2015_1280_MOESM12_ESM.tiff]
