# Supplementary material for: Involvement of a citrus meiotic recombination TTC-repeat motif in the formation of gross deletions generated by ionizing radiation and MULE activation
Source: BMC Genomics. 2015 Feb 13;16(1):69. doi: 10.1186/s12864-015-1280-3 (PMC4334395; doi:10.1186/s12864-015-1280-3)
Supplement: Additional file 5: Table S4. — CitMule Motif analysis performed with InterProScan. [file 12864_2015_1280_MOESM5_ESM.pdf]

Table S4. CitMule Motif analyses with InterProScan

Sequence "CitMule_1" crc64 checksum: F0DF841C7DEFB295 length: 795 aa.

InterPro IPR004330 FAR1 DNA binding domain			
method	AccNumber	shortName	location
HMMPfam	PF03101<br>6.7e-15	FAR1	T[34-118]

InterPro IPR006564 Zinc finger, PMZ-type<br>Molecular Function: zinc ion binding (GO:0008270)			
method	AccNumber	shortName	location
HMMSmart	SM00575<br>2.8e-08	plant mutator transposase zinc finger	T[532-559]

InterPro IPR007527 Zinc finger, SWIM-type<br>Molecular Function: zinc ion binding (GO:0008270)			
method	AccNumber	shortName	location
HMMPfam<br>1.6e-08	PF04434	SWIM	T[523-554]
ProfileScan<br>11.140	PS50966	ZF_SWIM	T[521-557]

InterPro IPR018289 MULE transposase domain			
method	AccNumber	shortName	location
HMMPfam<br>1.9e-27	PF10551	MULE	T[245-335]

Sequence "CitMule_2" crc64 checksum: 056D9725146CDB81 length: 795 aa.

InterPro IPR004330 FAR1 DNA binding domain			
method	AccNumber	shortName	location
HMMPfam	PF03101<br>4.6e-15	FAR1	T[34-118]

InterPro<br>Molecular Function: zinc ion binding (GO:0008270)	IPR006564	Zinc finger, PMZ-type	
method	AccNumber	shortName	location
HMMSmart	SM00575<br>2.8e-08	plant mutator transposase zinc finger	T[532-559]

InterPro<br>Molecular Function: zinc ion binding (GO:0008270)	IPR007527	Zinc finger, SWIM-type	
method	AccNumber	shortName	location
HMMPfam	PF04434<br>1.6e-08	SWIM	T[523-554]
ProfileScan	PS50966<br>11.140	ZF_SWIM	T[521-557]

InterPro	IPR018289	MULE transposase domain	
method	AccNumber	shortName	location
HMMPfam	PF10551<br>7.3e-28	MULE	T[245-335]

Sequence "CitMule_3" crc64 checksum: C9BC82AC2C535D52 length: 805 aa.

InterPro	IPR004330	FAR1 DNA binding domain	
method	AccNumber	shortName	location
HMMPfam	PF03101<br>6.8e-15	FAR1	T[34-118]

InterPro	IPR006564	Zinc finger, PMZ-type	
Molecular Function: zinc ion binding (GO:0008270)			
method	AccNumber	shortName	location
HMMSmart	SM00575<br>2.8e-08	plant mutator transposase zinc finger	T[532-559]

InterPro	IPR007527	Zinc finger, SWIM-type	
Molecular Function: zinc ion binding (GO:0008270)			
method	AccNumber	shortName	location
HMMPfam	PF04434<br>1.9e-08	SWIM	T[526-554]
ProfileScan	PS50966<br>11.214	ZF_SWIM	T[521-557]

InterPro	IPR018289	MULE transposase domain	
method	AccNumber	shortName	location
HMMPfam	PF10551<br>2e-27	MULE	T[245-335]

Sequence "CitMule_4" crc64 checksum: 17E170A04BCBEEAB length: 805 aa.

InterPro	IPR004330	FAR1 DNA binding domain	
method	AccNumber	shortName	location
HMMPfam	PF03101<br>6.8e-15	FAR1	T[34-118]

InterPro	IPR006564	Zinc finger, PMZ-type	
Molecular Function: zinc ion binding (GO:0008270)			
method	AccNumber	shortName	location
HMMSmart	SM00575<br>2.8e-08	plant mutator transposase zinc finger	T[532-559]

InterPro	IPR007527	Zinc finger, SWIM-type	
Molecular Function: zinc ion binding (GO:0008270)			
method	AccNumber	shortName	location
HMMPfam<br>1.7e-08	PF04434	SWIM	T[523-554]
ProfileScan	PS50966<br>11.140	ZF_SWIM	T[521-557]

InterPro	IPR018289	MULE transposase domain	
method	AccNumber	shortName	location
HMMPfam	PF10551<br>1.2e-27	MULE	T[245-335]
