# Supplementary material for: Involvement of a citrus meiotic recombination TTC-repeat motif in the formation of gross deletions generated by ionizing radiation and MULE activation
Source: BMC Genomics. 2015 Feb 13;16(1):69. doi: 10.1186/s12864-015-1280-3 (PMC4334395; doi:10.1186/s12864-015-1280-3)
Supplement: Additional file 6: Table S5. — Results of a MEGABLASTN search performed against the citrus ESTs of the GenBank. [file 12864_2015_1280_MOESM6_ESM.pdf]

Table S5. Sequences obtained with a MEGABLASTN search performed against the GenBank citrus ESTs

Acc. Nº	Clone name	Library	Query Citmule	% Identity	Alignment length	q. start	q. end	s. start	s. end	evalue
EY753043.1	CS00-C5-003-050-D05-CT.F	Sweet orange flower greenhouse plant Citrus sinensis cDNA	1 and 2	96.45	733	1263	1989	13	745	0.0
EY746647.1	CS00-C5-003-036-B01-CT.F	Sweet orange flower greenhouse plant Citrus sinensis	1 and 2	95.21	689	1269	1945	31	719	0.0
EY752157.1	23-508 CS00-C5-003-033-E03-CT.F	Sweet orange flower greenhouse plant Citrus sinensis cDNA	1 and 2	95.49	488	1263	1747	23	508	0.0
CX289210.1	C02010D11SK IF1	Citrus clementina cDNA clone C02010D11	1 and 2	100.00	422	1056	1477	1	422	0.0
EY710375.1	CS00-C3-701-107-B12-CT.F	Sweet orange fruit, development stadium (2 of 6) Citrus sinensis cDNA	1 and 2	87.27	479	1018	1487	33	509	3E-150
EY709068.1	CS00-C3-701-105-H04-CT.F	Sweet orange fruit, development stadium (2 of 6) Citrus sinensis cDNA	1 and 2	93.50	277	986	1260	11	286	6E-112
CX297718.1	C06004E04SK	RindPdig24 Citrus clementina cDNA clone C06004E04	1 and 2	100.00	113	2276	2388	1	113	7E-52
CX077986.1	UCRCS08_6C05_g	Parent Washington Navel Orange Callus cDNA Library UCRCS08-2 Citrus sinensis cDNA clone UCRCS08-6C05-E10-1-6.g	3 and 4	100.00	827	181	1007	829	3	0.0
CX044986.1	UCRCS07_20A05_b	Parent Washington Navel Orange Thrip-Challenged Flavedo cDNA Library UCRCS07 Citrus sinensis cDNA clone UCRCS07-20A05-A10-1-5.b	3 and 4	100.00	509	1778	2286	797	289	0.0
CN189940.1	UCRCS06_0002K16_f	Washington Navel Orange Stored Fruit Rind cDNA Library Citrus sinensis cDNA clone CS_WEc0002K16	3 and 4	100.00	234	2053	2286	544	311	4E-119
CK933199.1	CGF1004289_E03	Developing fruit juice sac at 38 DAFB Citrus sinensis cDNA clone JSJune0002_IVF_E03 5'	3 and 4	100.00	238	1	238	275	512	2E-121
CX077985.1	UCRCS08_6C05_b	Parent Washington Navel Orange Callus cDNA Library UCRCS08-2 Citrus sinensis cDNA clone UCRCS08-6C05-E10-1-5.b	3 and 4	100.00	201	1	201	658	858	8E-101
FC922781.1	KN0AAM1BD08RM1	SLH Citrus sinensis cDNA clone KN0AAM1BD08	3 and 4	100.00	133	2286	2418	675	543	5E-63
FE659144.1	C83	'Hong Anliu' sweet orange SSH library Citrus sinensis cDNA 5'	3 and 4	100.00	133	2286	2418	168	300	5E-63
EY698592.1	CS00-C3-700-073-E10-CT.F	Sweet orange fruit, development stadium (1 of 6) Citrus sinensis cDNA	3 and 4	100.00	133	2286	2418	690	558	5E-63
EY754961.1	CS12-C1-001-022-C08-CT.F	Sweet orange leaf, field plant A Citrus sinensis cDNA	3 and 4	100.00	133	2286	2418	651	519	5E-63
EY754580.1	CS12-C1-001-017-D11-CT.F	Sweet orange leaf, field plant A Citrus sinensis cDNA	3 and 4	100.00	133	2286	2418	651	519	5E-63
EY668713.1	CS00-C1-102-066-C08-CT.F	Sweet orange leaf, infected with Xylella fastidiosa (stage 2 of 2) Citrus sinensis cDNA	3 and 4	100.00	133	2286	2418	670	538	5E-63
EY754485.1	CS12-C1-001-016-B11-CT.F	Sweet orange leaf, field plant A Citrus sinensis cDNA	3 and 4	100.00	133	2286	2418	651	519	5E-63
EY671371.1	CS00-C1-102-080-D08-CT.F	Sweet orange leaf, infected with Xylella fastidiosa (stage 2 of 2) Citrus sinensis cDNA	3 and 4	100.00	133	2286	2418	674	542	5E-63
EX447666.1	Volcani_8_11_A2	Volcani08 Citrus reticulata x Citrus temple cDNA	3 and 4	100.00	133	2286	2418	588	456	5E-63
DN621587.1	UCRCS11_06O17_f	Parent Washington Navel Orange Scale-Infested Rind cDNA Library UCRCS11 Citrus sinensis cDNA clone CS_WEd0006O17	3 and 4	100.00	133	2286	2418	142	274	5E-63
CX672650.1	UCRCS10_18D03_g	Madame Vinous Sweet Orange Multiple Pathogen-Infected cDNA Library UCRCS10 Citrus sinensis cDNA clone UCRCS10-18D03-G6-6.g	3 and 4	100.00	133	2286	2418	671	539	5E-63
