# Supplementary material for: Involvement of a citrus meiotic recombination TTC-repeat motif in the formation of gross deletions generated by ionizing radiation and MULE activation
Source: BMC Genomics. 2015 Feb 13;16(1):69. doi: 10.1186/s12864-015-1280-3 (PMC4334395; doi:10.1186/s12864-015-1280-3)
Supplement: Additional file 11: Table S6. — Alternative allele frequency (± St dev) in the deletion identified in chromosome 3 of ARR and NER. Average allelic frequency based on Illumina reads (n > 10.000) and on PCR product sequencing (n = 16) after either direct amplification or after TA cloning are presented. [file 12864_2015_1280_MOESM11_ESM.pdf]

Table S6. Alternative allele frequency (± St dev) in the deletion identified in chromosome 3 of ARR and NER

Sequencing	CLE	ARR	NER
Illumina[a]	0.48± 0.1	0.81± 0.1	0.71± 0.1
Sanger[b]			
Young leaf			
Amplification	0.52 ± 0.1	0.79 ± 0.1	0.80 ± 0.1
TA cloning	0.58 ± 0.1	0.78 ± 0.1	0.85 ± 0.1
Fruit flavedo			
Amplification	0.54 ±0.0	0.78 ±0.1	0.75 ±0.1

[a] Average allelic frequency based on Illumina reads (n > 10000)

[b] Average allelic frequency based on after either direct amplification or after TA cloning
