# Supplementary material for: Involvement of a citrus meiotic recombination TTC-repeat motif in the formation of gross deletions generated by ionizing radiation and MULE activation
Source: BMC Genomics. 2015 Feb 13;16(1):69. doi: 10.1186/s12864-015-1280-3 (PMC4334395; doi:10.1186/s12864-015-1280-3)
Supplement: Additional file 13: Figure S7. — Sequence of the haploid CLE genome, the citrus reference genome (GenBank: AMZM00000000.1), delimiting a recombination motif detected inside the common deletion in chromosome 3 of ARR and NER. The position in red at 7797493 indicates the last single nucleotide variation (SNV) position in the mutants with prevalence of the alternative allele. The position at 7797753 marks the first SNV position with prevalence of the reference allele. The green stretch starting at position 7797537, between both red markers, indicates a putative recombination motif formed by 7 consecutive TTC trinucleotides. [file 12864_2015_1280_MOESM13_ESM.pdf]

genome scaffold_3:7796500-7798450

```
7796500 TTTTATTTAAGATTTACTTAAAATTTATTTTTATTTATTATCTATGATTT
    550 AACTAAATTGGTACTTTTACTTCTACTAACTCTTGAGATTAAATTTTCAT
    600 TACTTGTATATTTAAAAATTTATCAATAAAATTAACATTTAATAATTTAT
    650 TGCATTATTTGATTGTACTAAATTTAATAATCACTAATATAATATTAAAA
    700 AATAATCATGCAAATTATTAGCACCATTAATTAATTGATTCTATATAATA
    750 TTTTTAAATAATTGCTAAAAATTAATTAAATATTTAGATTGTTTTATGTA
    800 TTTTTATTTTATTATTATATTATGACTGCCCAATAAATTTAAATTTTCTT
    850 ATTAATTAATTTTTAAATTGATTTTCATTATGATTATAAAATTTTATGAA
    900 TAATTAAATATTATTTAAATATCAACTTAAATGCTATTTAATCTTATCTA
    950 AATATAATTTAATTTTACCAAAATAAGACTATAAAATATAATCAATCTTA
7797000 ATCCAATCTAACTTAATCCAGTCTAATTTTAATAATTAGTCCAATTCAAT
    050 TATACGTGCCAAACGTAACCTAATAGTCTTGATAAATTCCCCTTTGTGAA
    100 CTTATTGCTTTAACCTCAGTTTAAAATTACTACTTTAATCTTAGTTTAGT
    150 TACATGACAAGAAAAGAAAAAAAAAAAAGTTCTTTTTCTTGTTCACGCAT
    200 CACTCTCATCATTATTAAATACTGCAGTCGCTTGACTTTTTTGTTTCAAG
    250 TAAGTAATAAATAATCTTTTCCTTTCTTAATTCTTTAATTATTAGATAAT
    300 TTTAATATATTTTTTTTTCTATTTCAAATGGCAAAAGAAAGACAGAGAAT
    350 GTACAAGAATAGAGATTTTTTAAGAAAAAAAATTTCAAATAAAAGGAATA
    400 ATCCATTTAACAAATCAAAATGTCAAAAAGTTTAAGACAAAAATTGGGTG
    450 CAGATTACTAAATTCTATGGCCATCAAGATAATCAATCTTTTGCTTTTTT
    500 TTTATTGCTAGATTTTTGCTTCTTTATAGTAGATCGGTTCTTCTTCTTCT
    550 TCTTCTTCTGAGATAAACAATAATAAAGGAAAGCAATGGGGTTTAAAGAG
    600 GAGGGTTCATCGAGGGTTAGCCCTTGTAACTTATGTGATTTCAGATAATC
    650 AACAGACATAATACAACAGGCTTGAATTAATTCAAAGACACATGAATAAC
    700 AAGTCTTAATAAAAAAAAATAAAATGGGAAAGTCATTGAAGCTTGCTTGCT
    750 GTGCTTGCTCTGGTTTGCTTCTCCTGCCTTGTCAGCATCGCCGGTGCCCA
    800 AAACGGAAACGTACCCGTGGTGTGTGATAAATTTTATTATTGCAGCATTG
    850 ATTTTCAATATTGTACTAAAATTGATACCCGTGGTGCCTAAGTCAATGGA
    900 AGGAGGACAAAATTCTGCGGAGTAGAGCACTTGTTGCAGGTCGAAATTCT
    950 AATCTAACAATTGAAGCATTGGGACATAGGCCTAATTGTATTTGTTTCAT
7798000 TGATGAATGTGAACATGTGATGAATCTTTTTCGGATTTTGCTAATCCATG
    050 GGAATTTATAATCTCGAAGATGGAAGCATCAATGGTAAACATTTTTTGAG
    100 ACCCCATCAATCAATCCGTCTATGTGGGTGGAACAAATTAACTTTCCAGC
    150 ATCTTTATCAATCTATCATTTCATTGGTTTTTTAATTTATCAAATCATTA
    200 ATTTATTAACTTACCAATTATGGGTATTAATTTATTCACTGTTATACGCT
    250 ATTGTTCGGTACTCTTTAAACATCACCTCTTTGCTTGCTTACTCTAAAAA
    300 TAAATAATAATAATAATAATAAATGCAACATTAATCTTCTTTCAATATCA
    350 ACAATTTTTTTTTTTCAAACAAGGAAATTAGTAATAATTAAATGAAATCA
    400 CATAGAATAGACTCAATTTTTTTTTTCTTTCTTTCTTGAAGCAATCAACT
    450 GTATCGCTTAAAAAAATGTTTGTGAATGTGCTTCCATCTTCGATATTATA
```
