# Supplementary material for: Involvement of a citrus meiotic recombination TTC-repeat motif in the formation of gross deletions generated by ionizing radiation and MULE activation
Source: BMC Genomics. 2015 Feb 13;16(1):69. doi: 10.1186/s12864-015-1280-3 (PMC4334395; doi:10.1186/s12864-015-1280-3)
Supplement: Additional file 14: Table S7. — Listing of candidate genes tentatively associated with the observed ARR and NER phenotypic traits. [file 12864_2015_1280_MOESM14_ESM.pdf]

Table S7. Listing of candidate genes tentatively associated with the observed ARR and NER phenotypic traits

Seq name	Hit	Term	Phenotypic trait
Ciclev10024399m	S1 RNA-binding domain-containing protein	Chlorophyll biosynthetic process	Precociousness. Peel color change
Ciclev10020061m	Geranylgeranyl reductase	Chlorophyll biosynthetic process	Precociousness. Peel color change
Ciclev10019304m	Heparanase-like protein 1-like	Chlorophyll metabolic process<br>Starch and sucrose metabolic process	Precociousness. Peel color change<br>Precociousness. Brix accumulation
Ciclev10020378m	Malate chloroplastic-like	Carbohydrate metabolic processes<br>Reductive tricarboxylic acid cycle<br>Pyruvate metabolic process	Precociousness. Brix accumulation<br>Precociousness. Acid reduction<br>Precociousness. Acid reduction
Ciclev10020920m	Movement protein binding protein 2c	Sugar biosynthetic and catabolic process<br>Plasmodesmata transport	Precociousness. Brix accumulation<br>Precociousness. Fruit metabolism change
Ciclev10019047m	Transcriptional corepressor	Anatomical structure development	Precociousness
Ciclev10019317m	Replication protein a 70 kda DNA-binding subunit-like	Cell proliferation	Precociousness
Ciclev10020062m	Membrane bound o-acyl transferase family protein	Organ senescence	Precociousness
Ciclev10019953m	Phosphatidylinositol glycan anchor biosynthesis class u	GPI anchor biosynthetic process<br>Cell division	Precociousness
Ciclev10020101m	Flavin-containing monooxygenase fmo gs-ox-like 9-like	Phosphatidylinositol biosynthetic process	Precociousness
Ciclev10023002m	Ptd008 protein	Phosphatidylinositol biosynthetic process	Precociousness.
Ciclev10020097m	Protein cobra-like	Water transport, hyperosmotic response	Reduced tolerance to flooding
Ciclev10021527m	Protein cobra-like	Water transport, hyperosmotic response<br>Starch metabolic process	Reduced tolerance to flooding<br>Precociousness. Brix accumulation
Ciclev10021320m	Synaptosomal associated	Defense response to fungus	Reduced tolerance to fungus
Ciclev10022100m	Thaumatin-like protein	Defense response to fungus	Reduced tolerance to flooding
Ciclev10022104m	Thaumatin-like protein	Defense response to fungus	Reduced tolerance to flooding
Ciclev10023840m	Ap2 erf domain-containing transcription factor	Defense response to fungus	Reduced tolerance to flooding
Ciclev10024165m	Probable lrr receptor-like serine threonine-protein kinase at1g74360-like	Defense response to fungus	Reduced tolerance to flooding
