# Supplementary material for: Involvement of a citrus meiotic recombination TTC-repeat motif in the formation of gross deletions generated by ionizing radiation and MULE activation
Source: BMC Genomics. 2015 Feb 13;16(1):69. doi: 10.1186/s12864-015-1280-3 (PMC4334395; doi:10.1186/s12864-015-1280-3)
Supplement: Additional file 15: Table S8. — Specific primers for the determinations of gene dosage, allelic frequency (GD1) and chromosome rearrangement boundaries. Orientation: (+ forward, − reverse); start: position in reference genome. GD was used as control in gene dosage measurements. The transposon inversion found in Arrufatina was studied with ARR-5′ + CitMul_1 and ARR-CitMul_1 + 3′ primers. In Nero, NER-Del8-A, NER-Del8-B, NER-5′-T(8;6) and NER-3′-T(8;6) primers were used for the determination of deletion and transposition boundaries. [file 12864_2015_1280_MOESM15_ESM.pdf]

Table S8. Specific primers for the determinations of gene dosage, allelic frequency (GD1) and chromosome rearrangement boundaries

Name	Frame	Chr	Position	Sequence
GD[a]	+	3	8851426	5’-TCCTTGGATTCACGAGACCT-3’
	-	3	8851849	5’-GTTAAGGCTGCAAGGAAACG-3’
GD1a	+	3	7429651	5’-TGCCCATCATAACACAAAGC-3’
	-	3	7430170	5’-CAATTGGAAGGAGCAGAAGG-3’
GD2[a]	+	3	8053193	5’-TGCGAATGATACACGCAACT-3’
	-	3	8053552	5’-CAATCAAGGCTGCAATTTCC-3’
GD3[a]	+	3	8493139	5’-TTGAAGAAGCCTGGGGTAAG-3’
	-	3	8493454	5’-ACTTGCACCTGTTGGGAAAC-3’
ARR-5’+CitMul_1[b]	+	3	6785140	5’-GGTGGATTGGGAACCTACAG-3’
	-	3	6791802	5’-CATCCTTGTCCGGTAACACAC-3’
ARR-CitMul_1+3’[b]	+	3	6785486	5’-CTGGCTGCAATTGAACTCG-3’
	-	3	8686913	5’-AACTGTCACGGCTCAACCAT-3’
NER-Del8-A[b]	+	8	12601782	5’-ACGCCTCAGCTTGGTGATAC-3’
	-	8	13511756	5’-GTTCCGCAACTAGCTCCATC-3’
NER-Del8-B[b]	+	8	13566119	5’-TCAGATGCAAGAGCTTGTGAG-3’
	-	8	13636531	5’-CATGGGAATCAAGATGAGCA-3’
NER-5’-T(8;6)[b]	+	6	21997356	5’-ATTCATACCGGGCCCTAATC-3’
	-	8	13635261	5’-ATGCAGACTTTGCCGGTTAC-3’
NER-3’-T(8;6)[b]	+	8	13635106	5’-TTCATCCAAAGTGCTTGTGC-3’
	-	6	21998062	5’-GCGCTAGTGTTGAGTTGCTG-3’

[a] Gene dosage/allelic frequency
[b] Rearrangement boundaries
